# Anti-Inflammatory Effects of Lipid-Lowering Drugs and Supplements—A Narrative Review

**DOI:** 10.3390/nu15061517

**Published:** 2023-03-21

**Authors:** Stefan Zivkovic, Gorica Maric, Natasa Cvetinovic, Danijela Lepojevic-Stefanovic, Bojana Bozic Cvijan

**Affiliations:** 1 Department of Cardiovascular Disease, Zvezdara University Medical Center, 11000 Belgrade, Serbia; 2 Faculty of Medicine, Institute of Epidemiology, University of Belgrade, Dr. Subotica 8, 11000 Belgrade, Serbia; 3 Department of Cardiovascular Disease, University Medical Center “Dr Dragisa Misovic—Dedinje”, 11000 Belgrade, Serbia; 4 Department of Pharmacology, Clinical Pharmacology and Toxicology, Faculty of Medicine, University of Belgrade, 11000 Belgrade, Serbia

**Keywords:** inflammation, cardiovascular risk, atherosclerosis, immunomodulation, C-reactive protein, high-sensitivity C-reactive protein

## Abstract

Cardiovascular diseases (CVD) are the leading cause of death worldwide. Since the establishment of the “lipid hypothesis”, according to which, cholesterol level is directly correlated to the risk of CVD, many different lipid-lowering agents have been introduced in clinical practice. A majority of these drugs, in addition to their lipid-lowering properties, may also exhibit some anti-inflammatory and immunomodulatory activities. This hypothesis was based on the observation that a decrease in lipid levels occurs along with a decrease in inflammation. Insufficient reduction in the inflammation during treatment with lipid-lowering drugs could be one of the explanations for treatment failure and recurrent CVD events. Thus, the aim of this narrative review was to evaluate the anti-inflammatory properties of currently available lipid-lowering medications including statins, ezetimibe, bile acid sequestrants (BAS), proprotein convertase subtilisin/kexin type 9 (PCSK9) inhibitors, fibrates, omega-3 fatty acids, and niacin, as well as dietary supplements and novel drugs used in modern times.

## 1. Introduction

According to the World Health Organization’s (WHO) Global Health Estimates, ischemic heart disease (IHD) was the leading cause of death in 2019, accounting for 16% of all deaths worldwide [1]. Moreover, IHD was the condition with the highest increase in the number of deaths observed in the period from 2000 (2 million deaths) to 2019 (8.9 million deaths) [1]. Atherosclerosis is the main pathological process leading to the development of IHD, as well as many other cardiovascular diseases (CVD) such as carotid, vertebral, and renal artery stenosis; chronic ischemia of the lower limbs; chronic intestinal ischemia; etc. [2]. Atherosclerosis is characterized by the presence of cholesterol deposits in arterial walls; however, it is believed that the key feature of this process is chronic inflammation and that low-density lipoproteins (LDL) play a crucial role in its cascade. However, the association between LDL levels and risk for CVD occurrence is not yet completely understood given that in clinical practice, an increase in lipid levels is not always accompanied by a higher atherosclerotic burden and vice versa [3]. In that light, it has been shown that some other measures such as high-density lipoprotein (HDL) cholesterol and the level of HDL apolipoprotein AI and its ratio to apolipoprotein B (Apo B/A-I ratio) could represent better markers of atherosclerotic burden [3,4]. Despite this, it should be emphasized that data on the levels of different lipid parameters, including LDL, provide only partial information about the association between lipid profile and cardiovascular risk. In addition, lipoproteins may go through a range of modifications that alter their atherogenicity; however, measurement of these modified lipoproteins has not yet been introduced in routine clinical practice [5].

During the atherosclerotic process, cholesterol-rich particles penetrate the wall of arterial blood vessels, which results in subendothelial accumulation and retention of LDL, leading to their oxidization and/or modification [6]. Those oxidized LDL play a pivotal role in the formation of atherosclerotic plaque [7]. Based on the level of their oxidation, oxidized LDLs can be categorized as minimally modified LDL or extensively oxidized LDL [8]. In response to their presence, vascular smooth muscle cells produce mediators involved in the accumulation of monocytes. Oxidized LDL can also take part in this process. Following the recruitment of leukocytes and monocytes in the arterial wall, the next step in the atherosclerotic cascade is the transformation of monocytes into macrophage foam cells [7]. As a result, endothelial dysfunction occurs, resulting in the formation of atherosclerotic plaque, which consists of lipid particles, leukocytes, and calcium [9]. Their presence in the arterial wall is associated with the secretion of different immune mediators. So far, the role of inflammatory biomarkers such as interleukin (IL)-1β, tumor necrosis factor (TNF)-α, C-reactive protein (CRP), and IL-6 in the atherosclerotic process has been demonstrated [10,11,12,13]. As a result, systemic inflammation occurs, and the most commonly used indicator of its level is high-sensitivity CRP (hsCRP).

Since the establishment of the “lipid hypothesis” [14], according to which cholesterol level is directly correlated to the risk of CVD, many different lipid-lowering agents have been introduced in clinical practice. Interestingly, it has been shown that the majority of these drugs, in addition to their lipid-lowering properties, may also exhibit some anti-inflammatory and immunomodulatory activities. This hypothesis was based on the observation that a decrease in lipid levels occurs along with a decrease in inflammation. However, it is still not elucidated whether these changes in inflammation levels are driven by the lipid-lowering therapy itself or whether they are the result of LDL reduction and a consequent reverse in atherosclerosis. An insufficient reduction in inflammation during treatment with lipid-lowering drugs could be one of the explanations for treatment failure and recurrent CVD events [9]. In line with this, Tuñón et al., in their position paper [15], stated that the impact of the decrease in lipid levels on inflammation status is independent of the use of lipid-lowering therapy. On the other hand, the CANTOS trial tried to propose the proof of concept that modification of the inflammatory pathways themselves, specifically, the IL-6 signaling pathway, could impact the cardiovascular outcomes [13]. Therefore, the development of new treatment modalities with improved anti-inflammatory performances is an area of increasing interest in IHD.

Based on the aforementioned findings, the aim of the present narrative review is to evaluate the anti-inflammatory properties of currently available lipid-lowering medications.

## 2. Statins

Statins are the most commonly prescribed lipid-lowering drugs [16]. Currently, seven statins are available on the market: lovastatin, simvastatin, pravastatin, fluvastatin, atorvastatin, rosuvastatin, and pitavastatin [17]. They are usually divided into generations depending on their origin. Representatives of the first generation are isolated from fungal metabolites such as lovastatin, pravastatin, and simvastatin. Synthetic compounds such as atorvastatin, cerivastatin, fluvastatin, pitavastatin, and rosuvastatin represent statins of later generations [16]. Depending on their ability to dissolve in water or lipid-containing media, statins can be divided into hydrophilic (rosuvastatin and pravastatin) or lipophilic (simvastatin, fluvastatin, pitavastatin, lovastatin, atorvastatin) categories [18,19,20,21].

Due to safety concerns regarding the increased risk of rhabdomyolysis, cerivastatin is the only statin withdrawn from the worldwide markets [22].

The lipid-lowering mechanism of action of statins is well known. Statins are reversible and competitive inhibitors of the 3-hydroxy-3-methylglutaryl-coenzyme A (HMG-CoA) reductase enzyme. This enzyme is responsible for the conversion of 3-hydroxy-3-methylglutaryl-coenzyme A to mevalonate, the rate-limiting step in de novo cholesterol synthesis [23]. A consequence of HMG-CoA reductase enzyme inhibition is decreased mevalonate and cholesterol synthesis, which leads to a compensatory increase in the number of hepatic LDL receptors on the hepatocyte cell surface, uptake of LDL, and its precursors from circulation [16,23,24]. Additionally, statin consumption may inhibit the synthesis of apolipoprotein B-100 (apoB-100) and decrease the synthesis and secretion of triglyceride-rich particles [24,25]. Statins have a modest impact on increasing high-density lipoprotein cholesterol (HDL-C) concentration, while they have no effect on lipoprotein(a) (Lp(a)) concentration and size or density of LDL [25].

Studies have shown that the impact of statins on human health is beyond simple LDL lowering, which is also named “statin pleiotropy” [26,27]. So far, statins exhibited anti-inflammatory effects and benefits in diseases related to inflammation: atherosclerosis [28], chronic heart failure [29], sepsis [30], COVID-19 [31], diabetic nephropathy [32], gastric cancer [33,34], Alzheimer’s diseases [35], bone disorders [36], and autoimmune diseases [37]. Meta-analysis of 15 randomized controlled trials provided evidence that lipophilic statins pose more pleiotropic effects compared to hydrophilic statins due to easier penetration into the cell membranes [38].

Having in mind the association between elevated cholesterol and CVD, it is often difficult to separate the LDL-C-lowering effects of statins from their pleiotropic effects. Nevertheless, through the impact of statins on several factors of inflammation, beneficial statin effects independent of cholesterol reduction may be observed [39]. By considering the mechanism of action of statins, part of their pleotropic effects may be explained as well. Mevalonic acid is synthesized by HMG-CoA reductase and is the precursor of numerous metabolites such as the isoprenoid intermediates farnesylpyrophosphate (FPP) and geranylgeranylpyrophosphate (GGPP) [39,40]. When HMG-CoA reductase is blocked, decreased synthesis of isoprenoids and prenylation of small proteins are observed [40]. This evidence may be a possible explanation for the numerous anti-inflammatory effects of statins beyond their lipid-lowering characteristics.

The anti-inflammatory effects of statins can be observed through all the phases of formation, progression, and complications of atherosclerosis [41]. One of the first steps in atherosclerotic lesions is the accumulation of monocytes and macrophages in the arterial walls [42]. Statins can impact the adhesion and migration of inflammatory cells by diminishing the expression of integrin dimer CD11, vascular cell adhesion molecule (VCAM), and leukocyte-function antigen-1 (LFA-1) [42,43,44]. The next step in atherosclerosis is leukocyte migration to subendothelial sites in a process regulated by chemokines [41]. The protective role of statins in this step can be observed through reduced expression of the chemokine monocyte chemoattractant protein-1 (MCP-1), IL-8, which is regulated on activation by normally T-cell expressed and secreted (RANTES) on endothelial cells (ECs) and smooth muscle cells (SMCs) [45,46,47]. Another anti-inflammatory benefit of statins is their ability to reduce the expression of interferon (INF)-γ-induced major histocompatibility complex molecules class II (MHC II) [48].

So far, the correlation between increased levels of the inflammatory marker, CRP, and cardiovascular risk is well described [49]. CRP is an acute-phase reactant, and its production from hepatocytes is stimulated by IL-6, IL-1, and TNF-α [50]. Besides hepatocytes, there is evidence that CRP can be secreted locally by macrophages and artery smooth muscle cells involved in inflammation [51]. Numerous clinical trials have been designed to elucidate details of CRP and statin interaction nature and consequences [52,53,54,55,56]. Several mechanisms of statin-induced reduction in CRP have been proposed. One of the possibilities is the impact of statins on decreasing oxidized LDL (oxLDL) and, therefore, decreased concentration of inflammatory mediators [50]. Recently it has been proposed that the impact of statins on the serum apolipoprotein A-I (apoA-I) levels may be a contributing factor in reducing hsCRP levels [57]. Namely, by increasing apoA-I levels, statins decrease the expression of E-selectin, intracellular adhesion molecule-1 (ICAM-1), and VCAM-1 and consequently decrease inflammation [57]. Another proposed mechanism in combating local inflammation is decreased protein prenylation as a consequence of the ability of statins to decrease oxLDL [39]. As a result of decreased protein prenylation reduction in TNF and IL-6 levels is observed [58]. Interestingly, dual effects of statins were observed regarding the effect on IL-1 levels. On one side there are studies describing the ability of statins to decrease cytokine production such as IL-1 in monocytes in patients with hypercholesterolemia [59,60,61]. The other side of the medal is presented in studies that showed the ability of simvastatin to promote IL-1β activation [62,63]. So far, questions regarding the precise mechanism of statin and IL-1 expression remained open. Besides described mechanisms, even the possibility of direct interactions between statin molecules and CRP are suggested [64].

The impact of statins on nuceleotide-binding and oligomerization domain-like receptor family pyrin domain-containing 3 (NLRP3) inflammasome is under investigation. Studies have shown greater effects of lipophilic statins on NLRP3 complexes compared to hydrophilic statins due to their differences in chemistry and pharmacokinetics [39]. NLRP3 inflammasomes are cytosolic proteins, secreted by numerous immune cells and responsible for the activation of caspase-1 which releases IL-1β and IL-18 [65]. Nowadays, cholesterol is marked as an important NLRP3 activator [66]. Depending on the discussed representative of statin, dual impact, both activation or inhibition on NLRP3 has been described [39]. So far, several studies provided insight into molecular mechanisms of statins inflammasome activation including an increase in ATP release, ROS production, and lysosomal rupture [67,68,69]. On the other hand, the downregulation of NLRP3 by atorvastatin, and rosuvastatin is also stated [60,70,71].

Another possible anti-inflammatory mechanism of statins is their impact on the downregulation of nuclear factor-kappa B (NF-kB) and activator protein-1, transcription factors that influence inflammatory cytokines [39,72]. According to one of the hypotheses, the ability of statins to scavenge free oxygen radicals and to stimulate nitric oxide production leads to the stabilization of NF-kB inhibitor protein, IkBα [73,74]. During the years, studies in vitro, in vivo animal models, as well as in humans provided solid evidence regarding atorvastatin, fluvastatin, lovastatin, pravastatin, and simvastatin anti-inflammatory effects based on inhibition of NF-kB [75,76,77,78,79,80]. As a result of NF-kB inhibition, a shift to an anti-inflammatory response is obvious.

Another anti-inflammatory characteristic of statins that is independent of their lipid-lowering effects is their ability to decrease the expression of toll-like receptors (TLRs) 2 and 4 on immune cells and prevent lipopolysaccharide (LPS)-induced activation of monocytes, mononuclear cells, and endothelial cells [39,81]. As a result, the statins may lead to atherosclerotic plaque stabilization [39]. Depending on the analyzed study, several potential mechanisms on how statin impacts TLR-signaling pathways have been proposed: inhibition of protein prenylation, direct or indirect NF-kB inhibition, inhibition of MyD88/NF-kB pathway, enhancement of anti-inflammatory response elements [82,83,84,85,86]. Besides the previously discussed mechanisms, an additional mechanism of statin on inflammatory pathways is suggested, such as reduction in transforming growth factor (TGF)-β signaling in T cells, suppression of human dendritic cell maturation induced by oxLDL, disruption of T cell activation, and induction of T regulatory cells [87,88]. To unmask full molecular mechanisms and the multifaced anti-inflammatory nature of statins, further studies are needed.

## 3. Ezetimibe

Ezetimibe is an inhibitor of intestinal and biliary cholesterol absorption [24]. Ezetimibe inhibits cholesterol transport protein Niemann-Pick C1-like protein 1 (NPC1L1) at the level of the brush border of the small intestine [24,89,90]. As a consequence of decreased cholesterol delivery, the liver increases LDL receptor expression and clearance of LDL from the blood [24]. According to the latest European Guidelines on Dyslipidemia, in the case when LDL-C level is not achieved with the maximum tolerated dose of statin, a combination with ezetimibe is recommended [24]. An alleviating circumstance is the availability of ezetimibe generic products [91]. So far, ezetimibe has proved its efficiency in LDL-C reduction as a monotherapy [92] and in combination with statins [93], bile acid sequestrants [94], or bempedoic acid [24].

So far, studies have described the impact of ezetimibe monotherapy in reducing CRP as statistically nonsignificant compared with placebo [95,96]. A more effective reduction in CRP level was observed in a combination of ezetimibe with statins [96,97]. It was suggested that the ezetimibe effect on lowering CRP is not associated with improved anti-inflammatory function, and it may contribute to LDL reduction [98,99]. It was hypothesized that in order to achieve a decreased synthesis of inflammatory markers, such as CRP, a reduction in LDL of over 30% must be reached [97].

The effect of ezetimibe on several other parameters of inflammation was also investigated. It was shown that ezetimibe reduces the size of adipocytes, accumulation of pro-inflammatory cytokines, serum levels of free fatty acids, and the expression of the TNF-α [100,101]. Simvastatin and ezetimibe in combination reduced IL-18 levels and the expression of IL-1b [102]. A study that compared simvastatin, simvastatin/ezetimibe, and rosuvastatin at equivalent doses showed significant and similar reductions in plasma 8-Epi prostaglandin F2α (8-epiPGF2α), oxLDL, and lipoprotein-associated phospholipase A_2_ (Lp-PLA_2_) activity in patients with primary hypercholesterolemia [59]. Coadministration of simvastatin and ezetimibe resulted in a lower impact on transcription factor NF-kB binding activity compared to simvastatin monotherapy [103]. Considering that NF-kB activation is dependent not only on oxidated LDL cholesterol concentration but also on cytokines, free fatty acids, and molecules included in intracellular defense [103], this result may be additional proof of statin anti-inflammatory action beyond the lipid-lowering effects.

On the other hand, ezetimibe led to the degradation of IkB and, consequently, suppression of NF-kB activation via the mitogen-activated protein kinase (MAPK) pathway. These findings implied that there is a possibility of ezetimibe use in the therapy of inflammatory diseases [101]. The impact of ezetimibe on the Rho-associated coiled-coil-containing protein kinase (ROCK) was also monitored. It was suggested that statins reduced ROCK more significantly compared to statins and ezetimibe in combination [99].

The impact of ezetimibe on the endothelium was also investigated [104,105,106]. Several studies have investigated statin therapy versus statins with ezetimibe and have provided evidence of no difference in endothelial function [98,104,106,107,108,109,110]. It was suggested that in the absence of hypercholesterolemia, ezetimibe has no impact on endothelial function [104,108,109,110]. Furthermore, studies with better results on endothelial function achieved with statins alone were published [98,104,110]. These findings imply once again the possibility of a statin-specific pleiotropic anti-inflammatory effect. On the other hand, one comparative study described the beneficial effect on impaired endothelial function of atorvastatin and ezetimibe compared to atorvastatin alone [111]. It can be observed that ezetimibe may reduce inflammation in combination with statins, but the effect on endothelial function and its mechanisms remains unresolved.

## 4. Bile Acid Sequestrants (BAS)

BAS are traditional LDL-C-lowering drugs. They bind bile acids in the intestinal tract, increasing their fecal excretion and reducing enterohepatic circulation. As bile acids are the end products of cholesterol metabolism in the liver, the described process leads to increased bile acid synthesis and consequent serum LDL-C lowering and up-regulation of LDL receptors [112]. In addition, BAS glucose-lowering effect is very well established [113]. The three most frequently used medications from this group are cholestyramine, colestipol, and colesevelam. They can be used as monotherapy or in combination with other LDL-C lowering drugs. Recent large meta-analysis of 9 randomized trials and 1324 patients showed a 16.2% stronger reduction in LDL-C in patients treated with a statin and BAS than with statin alone [114]. The other pooled analysis of 3 randomized clinical trials of combined statin and colesevelam therapy demonstrated an additional 9.2% lowering of LDL-C in patients on combined therapy. The same analysis reported a significant hsCRP decrease (median change −23.3%) [115]. Colesevelam as monotherapy also demonstrated a reduction in hsCRP but failed to demonstrate IL-6 and TNF-α reduction [116]. One animal model suggests a possible BAS role in atherosclerosis stabilization in combination with brown fat activation [117].

Treatment with BAS can reduce the bioavailability of some anionic medications and vitamins [118]. However, colesevelam, because of its higher affinity for bile acids, can avoid these side effects [119]. It is not known whether their influence on the absorption of fat-soluble vitamins affects the potential anti-inflammatory properties of these medications. On the other hand, it is well established that high-dose vitamin E supplementation increases all-cause mortality [120], whereas paradoxically, supplementation with low doses in combination with other agents correlates with decreased all-cause mortality, while vitamin E in any dose combined with other agents also correlates with decreased mortality but only in disease-free populations [121]. These findings leave the issue of vitamin E supplementation in BAS treated patients still questionable.

## 5. Proprotein Convertase Subtilisin/Kexin Type 9 (PCSK9) Inhibitors

PCSK9 inhibitors are a heterogeneous group of medications used for the reduction in serum levels of LDL-C [24,122]. Currently, three agents from this group (alirocumab, evolocumab, and inclisiran) have received approval from the European Medicines Agency (EMA) and the U.S. Food and Drug Administration (USFDA) for the treatment of primary hypercholesterolemia or mixed dyslipidemia. Alirocumab and evolocumab are monoclonal antibodies for PCSK9 [122], while inclisiran is a synthetic small interfering RNA (siRNA) that, in a specific process, cleaves PCSK9 messenger RNA in the hepatocytes [123]. Both mechanisms lead to the up-regulation of LDL receptors and improved LDL clearance [123,124]. Several other agents that inhibit PCSK9 with similar or different mechanisms of action are under investigation, including anti-PCSK9 vaccines [125] and small oral anti-PCSK9 molecules [126]. Serum LDL-C reduction rates vary among trials investigating PCSK9 inhibitors on top of maximally tolerated statin therapy, but according to a recent large meta-analysis of 48 randomized trials, this rate is consistently between 50 and 65% concerning different agents and different dosage regiments [127]. Each of the three agents also significantly reduces the risk of cardiovascular events based on the FOURIER, the ODYSSEY OUTCOMES, and pooled analysis of the results of ORION-9, -10, and -11 trials [128,129,130].

Although PCSK9 inhibitors are relatively new medications, a substantial amount is already known about their possible pleiotropic effects, primarily anti-inflammatory effects. The association between inflammation, LDL-C levels, and atherosclerosis has been very well established. Moreover, there is evidence suggesting that PCSK9 levels are associated with the severity of coronary artery disease and positively correlate with the levels of inflammatory biomarkers including white blood cell, hsCRP, and fibrinogen [131]. Although traditional LDL-C-lowering drugs, especially statins, have shown a significant reduction in hsCRP, a similar relationship has not been demonstrated in randomized trials that investigated PCSK9 inhibitors [132,133,134]. Accurate mechanisms of anti-inflammatory effects of PCSK9 inhibition in atherosclerosis are still poorly elucidated. Most evidence is from experimental models and a small clinical trial with familial hypercholesterolemia patients, and they suggest impaired monocyte adherence and migration in the atherosclerotic plaque by reducing expression of the ICAM-1 in ECs and C-C chemokine receptor 2 (CCR2) in monocytes [135,136]. The last also showed down-regulation of pro-inflammatory TNF and up-regulation of anti-inflammatory IL-10 [136]. Furthermore, PCSK9 siRNA induces inhibition of PCSK9, and inflammatory mediators involved in the pathogenesis of atherosclerosis IL-1α, IL-6, and TNF-α in oxLDL-stimulated THP-1-derived macrophages via suppression of NF-κB nuclear translocation [137].

There are several studies based on different imaging methods aiming to support that PCSK9 inhibitors reduce arterial wall inflammation and atherosclerosis burden by modifying atherosclerotic plaque characteristics [138,139,140,141,142,143,144,145]. Initially, the ATHEROREMO-IVUS study using intravascular ultrasound virtual histology imaging for coronary atherosclerotic plaque characterization showed that PCSK9 levels were associated with the size of plaque necrotic core, even independently of serum LDL-C levels and concomitant statin therapy [138]. Afterward, many studies using different intravascular imaging techniques such as intravascular ultrasound, near-infrared spectroscopy, and optical coherence tomography almost unequivocally demonstrated that PCSK9 inhibitors positively affect all analyzed histological characteristics of atherosclerotic plaque including atheroma volume, lipid core burden index, fibrous cap thickness, lipid arc, macrophage accumulation, etc. [139,140,141,142]; only the ODYSSEY J-IVUS trial failed to confirm that treatment with alirocumab for 36 weeks after acute coronary syndrome reduces atheroma volume [143]. One small study that used nuclear magnetic resonance for evaluating carotid atherosclerotic plaque composition showed that treatment with alirocumab led to plaque stabilization [144]. Another small study investigating 18F-fluoro-2-deoxy-d-glucose (FDG) uptake in three large arteries (right and left carotid artery and aorta) demonstrated that long-term treatment with PCSK9 inhibitors reduces arterial FDG uptake independently of serum LDL-C levels [145].

There is rising evidence regarding the role of PCSK9 in other types of inflammation, including autoimmune diseases such as systemic lupus erythematosus (SLE), in which elevated serum levels of PCSK9 are associated with higher disease activity [146,147]; psoriasis, which is related to increased serum PCSK9 levels and higher PCSK9 expression in psoriatic lesions than in disease-free skin [148,149]; and HIV infection [150,151]. Data regarding sepsis and septic shock are conflicting. While some authors suggest that elevated PCSK9 levels in septic patients inhibit hepatocyte bacterial endotoxin clearance and promote multiple organ failure [152], the others point to higher mortality in patients with septic shock and lower PCSK9 levels [153], which is also supported by the results of a PCSK9 loss-of-function genotype study [154]. Initial clinical investigations with PCSK9 inhibitors in some of these conditions are promising. Recent studies also indicate elevated PCSK9 levels in many types of cancers, including gastric, colorectal, hepatocellular, breast, and thyroid cancers, and the potential role of PCSK9 in cancer biology [155,156]. Some beneficial effects of anti-PCSK9 immunization are suggested in experimental models of colorectal and breast cancer [157,158]. Moreover, data confirm that PCSK9 inhibition using alirocumab or evolocumab potentiates immune checkpoint inhibition therapy, specifically anti-PD1 antibody treatment, in mouse models of cancers [159]. Nonetheless, clinical data are lacking, and well-designed investigations in different cancer populations are required.

## 6. Novel LDL-C Lowering Drugs

The EMA and the USFDA approved three more LDL-C lowering drugs: bempedoic acid, as an alternative for the treatment of primary hypercholesterolemia or mixed dyslipidemia; lomitapide; and evinacumab for the treatment of homozygous familial hypercholesterolemia (HoFH). There is strong evidence supporting the LDL-C lowering efficacy of these medications; however, the data regarding their role in inflammation are still insufficient, and further investigations are warranted.

Pooled analysis of 4 CLEAR randomized trials of Bempedoic acid in patients with hypercholesterolemia and atherosclerotic cardiovascular disease (ASCVD) or with heterozygous familial hypercholesterolemia or with both on statin therapy or in statin-intolerant patients showed 17.8% LDL-C reduction in patients on a statin and 24.5% if they were statin-intolerant. Furthermore, the same analyses demonstrated a significant reduction in hsCRP (18.1% in patients on a statin and 27.4% in statin-intolerant patients), indicating a strong anti-inflammatory effect [160]. This effect is mainly related to the direct activation of AMP-activated protein kinase (AMPK). Initial animal model studies suggest a potential favorable impact of this drug on the pathogenesis of atherosclerosis [161,162]. Recently published results of the CLEAR outcome trial reported a beneficial effect of Bempedoic acid on the reduction in major adverse cardiovascular events, including death from cardiovascular causes, nonfatal myocardial infarction, nonfatal stroke, or coronary revascularization [163].

Treatment with lomitapide has led to significant LDL-C reduction (38% at week 78) in HoFH patients, according to a pivotal trial [164]. Long-term follow-up of these patients confirmed maintained lowering of LDL-C and, also, a progressive decrease in median hsCRP levels [165]. Observational studies of HoFH patients treated with lomitapide reported similar results indicating a steady reduction in LDL-C [166,167]. There are minimal data suggesting a protective lomitapide effect in ASCVD. One case series of six HoFH patients on long-term lomitapide therapy showed carotid intima-media thickness stabilization or regression [168]. In addition, recent studies emphasize the potential anti-cancer effects of lomitapide [169,170].

Records from the ELIPSE HoFH trial of evinacumab with combined lipid-lowering treatment shows a 49% LDL-C reduction compared to combined lipid-lowering treatment alone. A significant reduction was achieved in all analyzed secondary outcomes, including apolipoprotein B (apoB), TG, Lp(a), and apolipoprotein C-III (apoC-III) [171]. Similar trends have been obtained in a small real-world clinical practice trial [172]. On the other hand, there are no data confirming the role of evinacumab in inflammation so far, and data regarding atherosclerosis are rare but promising. Treatment with evinacumab reduced atherosclerotic lesion area and necrotic content in APOE*3Leiden.CETP mice [173]. In two patients treated with evinacumab, major atherosclerotic plaque regression was proven by using coronary computed tomography angiography (CCTA) [174]. Furthermore, there is strong evidence suggesting a relationship between ASCVD and high angiopoietin-like protein 3 (ANGPL3) levels [173,175,176].

## 7. Fibrates

Fibrates are fibric acid derivate agents, a type of amphipathic carboxylic acid that is currently widely used in patients with dyslipidemia. The USFDA approved the indications of fibrates for usage as an adjunct to dietary modifications in patients with primary hypercholesterolemia, mixed dyslipidemia, and severe hypertriglyceridemia. Recently, fenofibrate has emerged as a potential adjunct therapy for patients with primary biliary cholangitis who experience an inadequate response to standard therapy [177]. This is confirmed by a meta-analysis of 20 studies with 4783 participants [178], but it is still unapproved by the USFDA. A recent study showed that pemafibrate, which is fundamentally different in structure from other currently available fibrates, may lower incidence of nonalcoholic fatty liver disease [179]. Moreover, pemafibrate treatment in these patients is effective to control hepatic inflammation in the short term [180]. The latest European Society of Cardiology (ESC) guidelines recommended statin treatment as the first drug of choice for reducing CVD risk in high-risk adults with hypertriglyceridemia, while fibrates may be considered for usage in combination with statins in high-risk patients and in primary prevention in individuals who are at the target levels of LDL-C with elevated TG levels [24,181]. Although it is well known that fibrates have beneficial effects on lipid profile, they do not reduce CVD risk, which was recently confirmed in the PROMINENT clinical trial, which included more than ten thousand patients with high risk for CVD. Even though pemafibrate reduced TG, VLDL, and cholesterol remnant levels by 25–30%, incidence of cardiovascular events was not reduced [179].

Fibrates act by binding to the nuclear hormone receptor peroxisome proliferator-activated receptor (PPAR)-α. PPARα activation mediates changes in lipoprotein metabolism by inducing PPARα-dependent gene transcription, particularly by upregulating lipoprotein lipase, a key enzyme for TG-rich lipoprotein catabolism. Moreover, activation of PPARα reduces insulin resistance and dyslipidemia [182]. PPARα has a modulating effect on inflammation activity. Although it has been suggested that PPARα agonists may improve cardiac performance through anti-inflammatory effects [183], this assumption requires further evaluation. PPARα decreases the production of proinflammatory mediators (TNF-α, IL-1, IL-6, and IL-8) and modulates the expression of adhesive and chemotactic molecules. Furthermore, activation of PPARα can induce the production of anti-inflammatory agents, such as IL-10 [184]. Although under certain conditions, PPARα has pro-inflammatory effects, it is well-accepted that PPARα is involved primarily in anti-inflammatory signaling [183]. These effects of target receptor activation, as confirmed by in vitro and in vivo studies of both acute and chronic inflammatory processes [183], may elucidate the potential therapeutic use of fibrates in inflammatory disease. Furthermore, some fibrates (clofibrate, fenofibrate, gemfibrozil, and ciprofibrate) [185], as well as fibrate metabolites (clofibric and fenofibric acids), activate PPARγ, a participant in inflammatory reactions which inhibits the activation of immune cells and the expression of inflammatory factors [186,187]. It is proven that fenofibrate directly upregulates heme oxygenase-1, which contributes to the anti-inflammatory effects in human vascular cells [188]. Fenofibrate and clofibrate activate the gene coding for vanin-1, a protein with anti-inflammatory potential [189].

Fibrates have been evaluated in the context of many inflammatory states and diseases. In the randomized controlled trial that enrolled diabetic patients with mixed dyslipidemia, fenofibrate reduced levels of CRP by about 21–28% [190]. The anti-inflammatory effect of fenofibrate in metabolic syndrome is confirmed both in vitro and in an animal model [185,191]. What is more, fenofibrate improves colitis in IL-10-deficient animals, suggesting a possible therapeutic potential in inflammatory bowel disease [192]. Fenofibrate has potential applications both in therapy and for the prevention of bronchial remodeling in asthma [193,194], but that demands further research. The anti-inflammatory effects of fenofibrate have been proposed as a potential explanation of proven protection against diabetic retinopathy [195].

Pemafibrate, a novel selective PPARα modulator that was released in 2018, has superior binding efficiency to PPARα and a favorable safety profile over fenofibrate [196]. Due to these facts and evidence of beneficial effects on inflammation, pemfibrate deserves further evaluation in clinical settings.

Within the last few years, the majority of new synthetic fibrate derivatives have shown many biological effects such as hypolipidemic, hypoglycemic, anti-inflammatory, analgesic, antioxidant, and antiplatelet activities, which are mediated by, or independent of, PPAR activation. In light of their anti-inflammatory potential, amide-based fibrates have been evaluated in many preclinical studies; however, according to our knowledge, their effects have not been examined in clinical settings [197].

Although fibrates, as synthetic PPAR agonists, have effects on inflammation, which are well described and proven in preclinical studies, the benefits of these effects in humans need more clinical evidence. Given that fibrates are considered to be “one more lost paradise in lipid treatment” [198] and without evidence for CVD benefits [179], their anti-inflammatory effects represent an area of special interest, which deserves further elucidation.

## 8. Omega-3 Fatty Acids

The beneficial effects of omega-3 fatty acids in lipid metabolism regulation are well documented [199,200]. These polyunsaturated fatty acids are associated with decreased plasma TG levels, probably through inhibiting VLDL production [201]. They include alpha-linolenic acid (ALA), eicosapentaenoic acid (EPA), and docosahexaenoic acid (DHA), with the latter two being considered very-long-chain omega-3 fatty acids [202]. Although they can be found in plants, the most significant source of omega-3 fatty acids is fatty fish. Omega-3 and omega-6 polyunsaturated fatty acids represent constituents of the cell membrane. The predominance of omega-3 fatty acids is associated with less inflammatory conditions, while the predominance of omega-6 fatty acids leads to the promotion of inflammatory activity [203]. In line with this, in a systematic review conducted by Natto et al. [204], a relationship between omega-3 fatty acids and a decrease in the level of the following pro-inflammatory mediators was observed: apoB, apoA-I, total cholesterol (TC), and HDL-C. The same study also showed favorable effects of omega-3 fatty acids on TNF-α [204]. Besides their anti-inflammatory effects, omega-3 fatty acids are associated with antidysrhythmic, antiatherogenic, and antithrombotic activity, as well as with a decrease in systolic and diastolic blood pressure levels and overall improvement of endothelial activity [205].

Icosapent ethyl (IPE) is a highly purified ethyl ester of EPA [206]. The MARINE and the ANCHOR trials of IPE reported significant TG reduction in individuals with hypertriglyceridemia previously untreated or on statin therapy (33.1% and 21.5%, respectively) and led to the registration of this drug [207,208]. The same trials demonstrated significant differences in apoB levels between IPA and placebo groups after 12 weeks of treatment (9.1% in the MARINE trial and 8.8% in the ANCHOR trial) [207,208]. Several years later, the REDUCE-IT trial investigating cardiovascular effects of IPE in patients with hypertriglyceridemia and established CVD or diabetes and other risk factors, who were already on statin therapy, showed 25% relative and 4.8% absolute reduction in primary composite end point, including cardiovascular death, nonfatal myocardial infarction, nonfatal stroke, coronary revascularization, or unstable angina, a key secondary end point, and other tested end points, except death from any cause [209,210]. Similar trends were obtained in many prespecified subgroups, such as patients with a prior myocardial infarction, a prior percutaneous coronary intervention, or a previous coronary artery bypass graft (CABG) or with chronic kidney disease [211,212,213,214]. Furthermore, the REDUCE-IT biomarker sub-study showed a significant difference in serum levels of all tested inflammatory biomarkers (IL-1β, IL-6, hsCRP, oxLDL, homocysteine, Lp(a), lipoprotein-associated phospholipase A_2_ (Lp-PLA_2_)) at each time point [215]. The subsequent EVAPORATE trial, which enrolled 80 subjects with hypertriglyceridemia and multidetector-computed tomography (MDCS), confirmed that ASCVD demonstrated a clear benefit of IPE on atherosclerotic lesion stabilization proven by a reduction in low-attenuation plaque (LAP) volume and fibrous and fibro-fatty plaque volumes after 18 months of treatment [216]. This trial illuminated potential mechanisms behind the impressive results of the REDUCE-IT trial.

A recent study researching the effects of IPE on a rat model of ulcerative colitis has shown promising results in the reduction in different biohumoral, histologic, and immunohistochemical parameters of inflammation, oxidative stress, and apoptosis in colon tissue [217].

## 9. Lipid-Lowering Upplements

Currently, many different supplements are used for lipid-lowering purposes. Moreover, many combinations and dosage regiments are available. Anyway, their effectiveness and safety are usually not properly tested and vary significantly. In this study, we discuss results from several small, randomized trials investigating the anti-inflammatory effects of dietary LDL-C-lowering supplements. Red yeast rice monacolin K is probably the most frequently used agent from this group with the strongest evidence for its effectiveness. A recent large meta-analysis of 5868 participants using red yeast rice demonstrated significant LDL-C reduction [218]. Additionally, monacolin K showed a reduction in hsCRP levels in the population with untreated moderate hypercholesterolemia. The same trial demonstrated a decrease in levels of matrix metalloproteinases (MMPs) 2 and 9, which are considered markers of atherosclerotic plaque stability [219]. Moreover, monacolin K in nutraceutical combination with phytosterols, hydroxytyrosol, and vitamin E confirmed reduction in LDL-C and hsCRP levels in subjects with previously untreated hypercholesterolemia and low or moderate cardiovascular risk [220], while monacolin K-free combination did not demonstrate analogous results [221]. Ferulic acid supplementation reduced LDL-C, oxidized LDL-C, hsCRP, and TNF-α, as well as other lipids, oxidative stress, and inflammatory biomarkers in hyperlipidemic subjects [222]. Likewise, eight weeks of supplementation with barberry (Berberis integrrima) significantly lowered LDL-C, CRP, and IL-6 levels in individuals with cardiovascular risk factors [223]. These effects are potentially accomplished via the up-regulation of cell surface LDL receptor expression through the PCSK9 inhibition pathway [224]. New concepts of symbiotic supplementation showed some promising results in improving lipid profiles and decreasing inflammatory biomarkers in hemodialysis patients [225]. On the other hand, a recently published study of lipid-lowering and anti-inflammatory effects of different dietary supplements including fish oil, cinnamon, garlic, turmeric, plant sterols, and red yeast rice failed to demonstrate a significant reduction in LDL-C and hsCRP levels compared to placebo and low-dose Rosuvastatin in subjects with high 10-year risk for ASCVD after 28 days of treatment [226].

## 10. Conclusions

For a long time, cholesterol has been treated as the central point of ASCVD, and therapeutic modalities have been directed to its lowering. Nevertheless, evidence emerges that there is a significant interplay between lipid metabolism and immunity. The precise underlying mechanisms are yet to be discovered. Elucidating steps in this cascade is of particular interest considering the role of systemic inflammation in the formation of lipid plaques and atherosclerosis. On the other side, it has been demonstrated that, besides their impact on lipid levels, lipid-lowering drugs also express immunomodulatory activities by decreasing lipid levels and through lipid-metabolism-independent pathways (Table 1). It is still unknown how low lipid levels should be in order to achieve a satisfying decrease in the risk of CVD events and if it is also beneficial to include some anti-inflammatory drugs in the treatment. In this review, it was shown that statins demonstrate anti-inflammatory effects through multiple different mechanisms; however, it is still a matter of debate as to what extent these effects are driven by the drug itself. Positive anti-inflammatory effects of ezetimibe have been documented in animal models, as well as when it was administered in combination with statins. For PCSK9 inhibitors, there are some data suggesting possible alterations in inflammatory marker levels; however, further studies should provide deeper insight. Among omega-3 fatty acids, IPE offers promising results. On the other hand, the role of some lipid-lowering drugs such as BAS, fibrates, novel lipid-lowering drugs, and supplements in decreasing inflammation is questionable and requires further evaluation. Thus, improving knowledge on these interactions could help in tailoring treatments with lipid-lowering drugs and a consequent decrease in inflammation and the risk of new or recurrent CVD events. These observations offer possibilities for further interventions in the field of biomarkers of inflammation and the subsequent introduction of new lipid-lowering treatment modalities. Considering that CVD are attributed to the highest disease burden at the global level, even a small change in treatment effectiveness could have a large impact at the population level. Therefore, further studies in this area are warranted.

## Figures and Tables

**Table 1:** Impact of lipid-lowering drugs on selected inflammatory biomarkers.

Medication	Inflammatory Biomarkers				
	CRP or hsCRP	IL-1	IL-6	TNF	IL-10
Statins	↓	↑↓	↓	↓	/
Ezetimibe	↔	/	/	/	/
BAS	↓	/	↔	↔	/
PCSK9i	↔	↓	↓	↓	↑
Bempedoic acid	↓	/	/	/	/
Lomitapide	↓	/	/	/	/
Evinacumab	/	/	/	/	/
Fibrates	↓	↓	↓	↓	↑
Omega-3 fatty acids	/	/	/	↓	/
Icosapent ethyl	↓	↓	↓	/	/

↑—increased concentration; ↓—decreased concentration;/—no date available; ↔—no changes in the concentration; CRP—C—reactive protein; hsCRP—high-sensitivity C—reactive protein; IL-1—interleukin 1; IL-6—interleukin 6; TNF—tumor necrosis factor; IL-10—interleukin 10; BAS—bile acid sequestrants; PCSK9i—proprotein convertase subtilisin/kexin type 9 inhibitors.

## Data Availability

No new data were created or analyzed in this study. Data sharing is not applicable to this article.

## References

[1] WHO The Top 10 Causes of Death. https://www.who.int/news-room/fact-sheets/detail/the-top-10-causes-of-death.

[2] Jakubiak G.K., Pawlas N., Cieślar G., Stanek A. (2020). Chronic Lower Extremity Ischemia and Its Association with the Frailty Syndrome in Patients with Diabetes. Int. J. Environ. Res. Public Health.

[3] Kohsaka S., Jin Z., Rundek T., Homma S., Sacco R.L., Di Tullio M.R. (2010). Relationship between serum lipid values and atherosclerotic burden in the proximal thoracic aorta. Int. J. Stroke.

[4] Barter P., Gotto A.M., LaRosa J.C., Maroni J., Szarek M., Grundy S.M., Kastelein J.J., Bittner V., Fruchart J.C., Treating to New Targets Investigators (2007). HDL cholesterol, very low levels of LDL cholesterol, and cardiovascular events. N. Engl. J. Med..

[5] Feingold K.R., Feingold K.R., Anawalt B., Blackman M.R. (2023). Utility of Advanced Lipoprotein Testing in Clinical Practice.

[6] Bernardi S., Marcuzzi A., Piscianz E., Tommasini A., Fabris B. (2018). The Complex Interplay between Lipids, Immune System and Interleukins in Cardio-Metabolic Diseases. Int. J. Mol. Sci..

[7] Jebari-Benslaiman S., Galicia-García U., Larrea-Sebal A., Olaetxea J.R., Alloza I., Vandenbroeck K., Benito-Vicente A., Martín C. (2022). Pathophysiology of Atherosclerosis. Int. J. Mol. Sci..

[8] Parthasarathy S., Raghavamenon A., Garelnabi M.O., Santanam N., Uppu R.M., Murthy S.N., Pryor W.A., Parinandi N.L. (2010). Oxidized Low-Density Lipoprotein. Free Radicals and Antioxidant Protocols.

[9] Ugovšek S., Zupan J., Rehberger Likozar A., Šebeštjen M. (2021). Influence of lipid-lowering drugs on inflammation: What is yet to be done?. Arch. Med. Sci..

[10] Stopeck A.T., Nicholson A.C., Mancini F.P., Hajjar D.P. (1993). Cytokine regulation of low density lipoprotein receptor gene transcription in HepG2 cells. J. Biol. Chem..

[11] Jialal I., Devaraj S., Venugopal S.K. (2004). C-reactive protein: Risk marker or mediator in atherothrombosis?. Hypertension.

[12] Chang M.K., Binder C.J., Torzewski M., Witztum J.L. (2002). C-reactive protein binds to both oxidized LDL and apoptotic cells through recognition of a common ligand: Phosphorylcholine of oxidized phospholipids. Proc. Natl. Acad. Sci. USA.

[13] Ridker P.M., Libby P., MacFadyen J.G., Thuren T., Ballantyne C., Fonseca F., Koenig W., Shimokawa H., Everett B.M., Glynn R.J. (2018). Modulation of the interleukin-6 signalling pathway and incidence rates of atherosclerotic events and all-cause mortality: Analyses from the Canakinumab Anti-Inflammatory Thrombosis Outcomes Study (CANTOS). Eur. Heart J..

[14] Steinberg D. (2005). Thematic review series: The pathogenesis of atherosclerosis. An interpretive history of the cholesterol controversy: Part, I.I. the early evidence linking hypercholesterolemia to coronary disease in humans. J. Lipid Res..

[15] Tuñón J., Badimón L., Bochaton-Piallat L., Cariou B., Daemen M.J., Egido J., Evans P.C., Hoefer I.E., Ketelhuth J.D.F., Lutgens E. (2019). Identifying the anti-inflammatory response to lipid lowering therapy: A position paper from the working group on atherosclerosis and vascular biology of the European Society of Cardiology. Cardiovasc. Res..

[16] Schachter M. (2005). Chemical, pharmacokinetic and pharmacodynamic properties of statins: An update. Fundam. Clin. Pharmacol..

[17] Pahan K. (2006). Lipid-lowering drugs. Cell. Mol. Life Sci..

[18] Climent E., Benaiges D., Pedro-Botet J. (2021). Hydrophilic or Lipophilic Statins?. Front. Cardiovasc. Med..

[19] Endo A., Kuroda M., Tsujita Y. (1976). ML-236A, ML-236B, and ML-236C, new inhibitors of cholesterogenesis produced by Penicillium citrinium. J. Antibiot..

[20] Alberts A.W., Chen J., Kuron G., Hunt V., Huff J., Hoffman C., Rothrock J., Lopez M., Joshua H., Harris E. (1980). Mevinolin: A highly potent competitive inhibitor of hydroxymethylglutaryl-coenzyme A reductase and a cholesterol-lowering agent. Proc. Natl. Acad. Sci. USA.

[21] Harrington R.A. (2017). Statins—Almost 30 Years of Use in the United States and Still Not Quite There. JAMA Cardiol..

[22] Davidson M.H. (2002). Controversy surrounding the safety of cerivastatin. Expert Opin. Drug Saf..

[23] Almuti K., Rimawi R., Spevack D., Ostfeld R.J. (2006). Effects of statins beyond lipid lowering: Potential for clinical benefits. Int. J. Cardiol..

[24] Mach F., Baigent C., Catapano A.L., Koskinas K.C., Casula M., Badimon L., Chapman M.J., De Backer G.G., Delgado V., Ference B.A. (2020). 2019 ESC/EAS Guidelines for the management of dyslipidaemias: Lipid modification to reduce cardiovascular risk. Eur. Heart J..

[25] Maron D.J., Fazio S., Linton M.F. (2000). Current perspectives on statins. Circulation.

[26] Marzilli M. (2010). Pleiotropic effects of statins: Evidence for benefits beyond LDL-cholesterol lowering. Am. J. Cardiovasc. Drugs.

[27] Yu D., Liao J.K. (2022). Emerging views of statin pleiotropy and cholesterol lowering. Cardiovasc. Res..

[28] Ross R. (1999). Atherosclerosis—An inflammatory disease. N. Engl. J. Med..

[29] Kandelouei T., Abbasifard M., Imani D., Aslani S., Razi B., Fasihi M., Shafiekhani S., Mohammadi K., Jamialahmadi T., Reiner Ž. (2022). Effect of Statins on Serum level of hs-CRP and CRP in Patients with Cardiovascular Diseases: A Systematic Review and Meta-Analysis of Randomized Controlled Trials. Mediat. Inflamm..

[30] Merx M.W., Liehn E.A., Janssens U., Lütticken R., Schrader J., Hanrath P., Weber C. (2004). HMG-CoA reductase inhibitor simvastatin profoundly improves survival in a murine model of sepsis. Circulation.

[31] Santosa A., Franzén S., Nåtman J., Wettermark B., Parmryd I., Nyberg F. (2022). Protective effects of statins on COVID-19 risk, severity and fatal outcome: A nationwide Swedish cohort study. Sci. Rep..

[32] Sun H., Yuan Y., Sun Z.L. (2013). Cholesterol Contributes to Diabetic Nephropathy through SCAP-SREBP-2 Pathway. Int. J. Endocrinol..

[33] Su C.H., Islam M.M., Jia G., Wu C.C. (2022). Statins and the Risk of Gastric Cancer: A Systematic Review and Meta-Analysis. J. Clin. Med..

[34] Kwon M.J., Kang H.S., Kim J.H., Kim J.H., Kim S.H., Kim N.Y., Nam E.S., Min K.W., Choi H.G. (2021). Association between Statin Use and Gastric Cancer: A Nested Case-Control Study Using a National Health Screening Cohort in Korea. Pharmaceuticals.

[35] Fonseca A.C., Resende R., Oliveira C.R., Pereira C.M. (2010). Cholesterol and statins in Alzheimer’s disease: Current controversies. Exp. Neurol..

[36] Chamani S., Liberale L., Mobasheri L., Montecucco F., Al-Rasadi K., Jamialahmadi T., Sahebkar A. (2021). The role of statins in the differentiation and function of bone cells. Eur. J. Clin. Investig..

[37] Li X.L., Dou Y.C., Liu Y., Shi C.W., Cao L.L., Zhang X.Q., Zhu J., Duan R.S. (2011). Atorvastatin ameliorates experimental autoimmune neuritis by decreased Th1/Th17 cytokines and up-regulated T regulatory cells. Cell. Immunol..

[38] Sahebkar A., Kotani K., Serban C., Ursoniu S., Mikhailidis D.P., Jones S.R., Ray K.K., Blaha M.J., Rysz J., Toth P.P. (2015). Statin therapy reduces plasma endothelin-1 concentrations: A meta-analysis of 15 randomized controlled trials. Atherosclerosis.

[39] Koushki K., Shahbaz S.K., Mashayekhi K., Sadeghi M., Zayeri Z.D., Taba M.Y., Banach M., Al-Rasadi K., Johnston T.P., Sahebkar A. (2021). Anti-inflammatory Action of Statins in Cardiovascular Disease: The Role of Inflammasome and Toll-Like Receptor Pathways. Clin. Rev. Allergy Immunol..

[40] Tricarico P.M., Crovella S., Celsi F. (2015). Mevalonate Pathway Blockade, Mitochondrial Dysfunction and Autophagy: A Possible Link. Int. J. Mol. Sci..

[41] Schönbeck U., Libby P. (2004). Inflammation, immunity, and HMG-CoA reductase inhibitors: Statins as antiinflammatory agents?. Circulation.

[42] Rasmussen L.M., Hansen P.R., Nabipour M.T., Olesen P., Kristiansen M.T., Ledet T. (2001). Diverse effects of inhibition of 3-hydroxy-3-methylglutaryl-CoA reductase on the expression of VCAM-1 and E-selectin in endothelial cells. Biochem. J..

[43] Weber C., Erl W., Weber K.S., Weber P.C. (1997). HMG-CoA reductase inhibitors decrease CD11b expression and CD11b-dependent adhesion of monocytes to endothelium and reduce increased adhesiveness of monocytes isolated from patients with hypercholesterolemia. J. Am. Coll. Cardiol..

[44] Weitz-Schmidt G., Welzenbach K., Brinkmann V., Kamata T., Kallen J., Bruns C., Cottens S., Takada Y., Hommel U. (2001). Statins selectively inhibit leukocyte function antigen-1 by binding to a novel regulatory integrin site. Nat. Med..

[45] Romano M., Diomede L., Sironi M., Massimiliano L., Sottocorno M., Polentarutti N., Guglielmotti A., Albani D., Bruno A., Fruscella P. (2000). Inhibition of monocyte chemotactic protein-1 synthesis by statins. Lab. Investig..

[46] Diomede L., Albani D., Sottocorno M., Donati M.B., Bianchi M., Fruscella P., Salmona M. (2001). In vivo anti-inflammatory effect of statins is mediated by nonsterol mevalonate products. Arterioscler. Thromb. Vasc. Biol..

[47] Jougasaki M., Ichiki T., Takenoshita Y., Setoguchi M. (2010). Statins suppress interleukin-6-induced monocyte chemo-attractant protein-1 by inhibiting Janus kinase/signal transducers and activators of transcription pathways in human vascular endothelial cells. Br. J. Pharmacol..

[48] Lee S.J., Qin H., Benveniste E.N. (2008). The IFN-γ-Induced Transcriptional Program of the CIITA Gene Is Inhibited by Statins. Eur. J. Immunol..

[49] Pai J.K., Pischon T., Ma J., Manson J.E., Hankinson S.E., Joshipura K., Curhan G.C., Rifai N., Cannuscio C.C., Stampfer M.J. (2004). Inflammatory markers and the risk of coronary heart disease in men and women. N. Engl. J. Med..

[50] Arévalo-Lorido J.C. (2016). Clinical relevance for lowering C-reactive protein with statins. Ann. Med..

[51] Calabró P., Willerson J.T., Yeh E.T. (2003). Inflammatory cytokines stimulated C-reactive protein production by human coronary artery smooth muscle cells. Circulation.

[52] Nissen S.E., Tuzcu E.M., Schoenhagen P., Brown B.G., Ganz P., Vogel R.A., Crowe T., Howard G., Cooper C.J., Brodie B. (2004). Effect of intensive compared with moderate lipid-lowering therapy on progression of coronary atherosclerosis: A randomized controlled trial. JAMA.

[53] Puri R., Nissen S.E., Libby P., Shao M., Ballantyne C.M., Barter P.J., Chapman M.J., Erbel R., Raichlen J.S., Uno K. (2013). C-reactive protein, but not low-density lipoprotein cholesterol levels, associate with coronary atheroma regression and cardiovascular events after maximally intensive statin therapy. Circulation.

[54] Hong Y.J., Jeong M.H., Ahn Y., Kim S.W., Bae J.H., Hur S.H., Ahn T.H., Rha S.W., Kim K.S., Chae I.H. (2012). Effect of pitavastatin treatment on changes of plaque volume and composition according to the reduction of high-sensitivity C-reactive protein levels. J. Cardiol..

[55] Noguchi T., Tanaka A., Kawasaki T., Goto Y., Morita Y., Asaumi Y., Nakao K., Fujiwara R., Nishimura K., Miyamoto Y. (2015). Effect of Intensive Statin Therapy on Coronary High-Intensity Plaques Detected by Noncontrast T1-Weighted Imaging: The AQUAMARINE Pilot Study. J. Am. Coll. Cardiol..

[56] Komukai K., Kubo T., Kitabata H., Matsuo Y., Ozaki Y., Takarada S., Okumoto Y., Shiono Y., Orii M., Shimamura K. (2014). Effect of atorvastatin therapy on fibrous cap thickness in coronary atherosclerotic plaque as assessed by optical coherence tomography: The EASY-FIT study. J. Am. Coll. Cardiol..

[57] Tani S., Takahashi A., Nagao K., Hirayama A. (2015). Contribution of apolipoprotein A-I to the reduction in high-sensitivity C-reactive protein levels by different statins: Comparative study of pitavastatin and atorvastatin. Heart Vessel..

[58] Greenwood J., Steinman L., Zamvil S.S. (2006). Statin therapy and autoimmune disease: From protein prenylation to immunomod-ulation. Nat. Rev. Immunol..

[59] Moutzouri E., Tellis C.C., Rousouli K., Liberopoulos E.N., Milionis H.J., Elisaf M.S., Tselepis A.D. (2012). Effect of simvastatin or its combination with ezetimibe on Toll-like receptor expression and lipopolysaccharide-induced cytokine production in monocytes of hypercholesterolemic patients. Atherosclerosis.

[60] Altaf A., Qu P., Zhao Y., Wang H., Lou D., Niu N. (2015). NLRP3 inflammasome in peripheral blood monocytes of acute coronary syndrome patients and its relationship with statins. Coron. Artery Dis..

[61] Loppnow H., Zhang L., Buerke M., Lautenschläger M., Chen L., Frister A., Schlitt A., Luther T., Song N., Hofmann B. (2011). Statins potently reduce the cytokine-mediated IL-6 release in SMC/MNC cocultures. J. Cell. Mol. Med..

[62] Massonnet B., Normand S., Moschitz R., Delwail A., Favot L., Garcia M., Bourmeyster N., Cuisset L., Grateau G., Morel F. (2009). Pharmacological inhibitors of the mevalonate pathway activate pro-IL-1 processing and IL-1 release by human monocytes. Eur. Cytokine Netw..

[63] Kuijk L.M., Mandey S.H., Schellens I., Waterham H.R., Rijkers G.T., Coffer P.J., Frenkel J. (2008). Statin synergizes with LPS to induce IL-1beta release by THP-1 cells through activation of caspase-1. Mol. Immunol..

[64] Shakour N., Ruscica M., Hadizadeh F., Cirtori C., Banach M., Jamialahmadi T., Sahebkar A. (2020). Statins and C-reactive protein: In silico evidence on direct interaction. Arch. Med. Sci..

[65] Blevins H.M., Xu Y., Biby S., Zhang S. (2022). The NLRP3 Inflammasome Pathway: A Review of Mechanisms and Inhibitors for the Treatment of Inflammatory Diseases. Front. Aging Neurosci..

[66] Duewell P., Kono H., Rayner K.J., Sirois C.M., Vladimer G., Bauernfeind F.G., Abela G.S., Franchi L., Nuñez G., Schnurr M. (2010). NLRP3 inflammasomes are required for atherogenesis and activated by cholesterol crystals. Nature.

[67] Liao Y.H., Lin Y.C., Tsao S.T., Lin Y.C., Yang A.J., Huang C.T., Huang K.C., Lin W.W. (2013). HMG-CoA reductase inhibitors activate caspase-1 in human monocytes depending on ATP release and P2X7 activation. J. Leukoc. Biol..

[68] Xu J.F., Washko G.R., Nakahira K., Hatabu H., Patel A.S., Fernandez I.E., Nishino M., Okajima Y., Yamashiro T., Ross J.C. (2012). Statins and pulmonary fibrosis: The potential role of NLRP3 inflammasome activation. Am. J. Respir. Crit. Care Med..

[69] Henriksbo B.D., Lau T.C., Cavallari J.F., Denou E., Chi W., Lally J.S., Crane J.D., Duggan B.M., Foley K.P., Fullerton M.D. (2014). Fluvastatin causes NLRP3 inflammasome-mediated adipose insulin re-sistance. Diabetes.

[70] Davaro F., Forde S.D., Garfield M., Jiang Z., Halmen K., Tamburro N.D., Kurt-Jones E., Fitzgerald K.A., Golenbock D.T., Wang D. (2014). 3-Hydroxyl-3-methylglutaryl coenzyme A (HMG-CoA) reductase inhibitor (statin)-induced 28-kDa interleukin-1β interferes with mature IL-1β signaling. J. Biol. Chem..

[71] Luo B., Li B., Wang W., Liu X., Liu X., Xia Y., Zhang C., Zhang Y., Zhang M., An F. (2014). Rosuvastatin alleviates diabetic cardiomyopathy by inhibiting NLRP3 inflammasome and MAPK pathways in a type 2 diabetes rat model. Cardiovasc. Drugs Ther..

[72] Dichtl W., Dulak J., Frick M., Alber H.F., Schwarzacher S.P., Ares M.P., Nilsson J., Pachinger O., Weidinger F. (2003). HMG-CoA reductase inhibitors regulate inflammatory transcription factors in human endothelial and vascular smooth muscle cells. Arterioscler. Thromb. Vasc. Biol..

[73] Peng H.B., Libby P., Liao J.K. (1995). Induction and stabilization of IκB alpha by nitric oxide mediates inhibition of NF-κB. J. Biol. Chem..

[74] Yamamoto A., Hoshi K., Ichihara K. (1998). Fluvastatin, an inhibitor of 3-hydroxy-3-methylglutaryl-CoA reductase, scavenges free radicals and inhibits lipid peroxidation in rat liver microsomes. Eur. J. Pharmacol..

[75] Ortego M., Bustos C., Hernández-Presa M.A., Tuñón J., Díaz C., Hernández G., Egido J. (1999). Atorvastatin reduces NF-κB activation and chemokine expression in vascular smooth muscle cells and mononuclear cells. Atherosclerosis.

[76] Hernández-Presa M.A., Martín-Ventura J.L., Ortego M., Gómez-Hernández A., Tuñón J., Hernández-Vargas P., Blanco-Colio L.M., Mas S., Aparicio C., Ortega L. (2002). Atorvastatin reduces the expression of cyclooxygenase-2 in a rabbit model of atherosclerosis and in cultured vascular smooth muscle cells. Atherosclerosis.

[77] Chandrasekar B., Mummidi S., Mahimainathan L., Patel D.N., Bailey S.R., Imam S.Z., Greene W.C., Valente A.J. (2006). Interleukin-18-induced human coronary artery smooth muscle cell migration is dependent on NF-κB- and AP-1-mediated matrix metalloproteinase-9 expression and is inhibited by atorvastatin. J. Biol. Chem..

[78] Guijarro C., Kim Y., Schoonover C.M., Massy Z.A., O’Donnell M.P., Kasiske B.L., Keane W.F., Kashtan C.E. (1996). Lovastatin inhibits lipopolysaccharide-induced NF-κB activation in human mesangial cells. Nephrol. Dial. Transplant..

[79] Ozbek E., Cekmen M., Ilbey Y.O., Simsek A., Polat E.C., Somay A. (2009). Atorvastatin prevents gentamicin-induced renal damage in rats through the inhibition of p38-MAPK and NF-κB pathways. Ren. Fail..

[80] Sheridan A., Wheeler-Jones C.P.D., Gage M.C. (2022). The Immunomodulatory Effects of Statins on Macrophages. Immuno.

[81] Kapelouzou A., Giaglis S., Peroulis M., Katsimpoulas M., Moustardas P., Aravanis C.V., Kostakis A., Karayannakos P.E., Cokkinos D.V. (2017). Overexpression of Toll-Like Receptors 2, 3, 4, and 8 is Correlated to the Vascular Atherosclerotic Process in the Hyperlipidemic Rabbit Model: The Effect of Statin Treatment. J. Vasc. Res..

[82] Bruiners N., Dutta N.K., Guerrini V., Salamon H., Yamaguchi K.D., Karakousis P.C., Gennaro M.L. (2020). The anti-tubercular activity of simvastatin is mediated by cholesterol-driven autophagy via the AMPK-mTORC1-TFEB axis. J. Lipid Res..

[83] Han F., Xiao Q.Q., Peng S., Che X.Y., Jiang L.S., Shao Q., He B. (2018). Atorvastatin ameliorates LPS-induced inflammatory response by autophagy via AKT/mTOR signaling pathway. J. Cell. Biochem..

[84] Abe M., Matsuda M., Kobayashi H., Miyata Y., Nakayama Y., Komuro R., Fukuhara A., Shimomura I. (2008). Effects of Statins on Adipose Tissue Inflammation: Their Inhibitory Effect on MyD88-Independent IRF3/IFN-β Pathway in Macrophages. Arterioscler. Thromb. Vasc. Biol..

[85] Brosseau C., Colas L., Magnan A., Brouard S. (2018). CD9 Tetraspanin: A New Pathway for the Regulation of Inflamma-tion?. Front. Immunol..

[86] Suzuki M., Tachibana I., Takeda Y., He P., Minami S., Iwasaki T., Kida H., Goya S., Kijima T., Yoshida M. (2009). Tetraspanin CD9 Negatively Regulates Lipopolysaccharide-Induced Macrophage Activation and Lung Inflammation. J. Immunol..

[87] Gojova A., Brun V., Esposito B., Cottrez F., Gourdy P., Ardouin P., Tedgui A., Mallat Z., Groux H. (2003). Specific abrogation of transforming growth factor-beta signaling in T cells alters atherosclerotic lesion size and composition in mice. Blood.

[88] Frostegård J., Zhang Y., Sun J., Yan K., Liu A. (2016). Oxidized Low-Density Lipoprotein (OxLDL)-Treated Dendritic Cells Promote Activation of T Cells in Human Atherosclerotic Plaque and Blood, Which Is Repressed by Statins: MicroRNA let-7c Is Integral to the Effect. J. Am. Heart Assoc..

[89] Arsenault B.J., Perroti N., Puri R. (2018). Therapeutic Agents Targeting Cardiometabolic Risk for Preventing and Treating Atherosclerotic Cardiovascular Diseases. Clin. Pharmacol. Ther..

[90] Tobaru T., Seki A., Asano R., Sumiyoshi T., Hagiwara N. (2013). Lipid-lowering and anti-inflammatory effect of ezetimibe in hyperlipidemic patients with coronary artery disease. Heart Vessel..

[91] Palmer J.L., Kunhihitlu A., Costantini A., Esquivel F., Roush J., Edwards K., Hill T.W. (2014). Pharmacokinetic bioequivalence crossover study of branded generic and innovator formulations of the cholesterol lowering agent ezetimibe. Clin. Pharmacol. Drug Dev..

[92] Pandor A., Ara R.M., Tumur I., Wilkinson A.J., Paisley S., Duenas A., Durrington P.N., Chilcott J. (2009). Ezetimibe monotherapy for cholesterol lowering in 2722 people: Systematic review and meta-analysis of randomized controlled trials. J. Intern. Med..

[93] Morrone D., Weintraub W.S., Toth P.P., Hanson M.E., Lowe R.S., Lin J., Shah A.K., Tershakovec A.M. (2012). Lipid-altering efficacy of ezetimibe plus statin and statin monotherapy and identification of factors associated with treatment response: A pooled analysis of over 21,000 subjects from 27 clinical trials. Atherosclerosis.

[94] Bays H., Rhyne J., Abby S., Lai Y.L., Jones M. (2006). Lipid-lowering effects of colesevelam HCl in combination with ezetimibe. Curr. Med. Res. Opin..

[95] Sager P.T., Melani L., Lipka L., Strony J., Yang B., Suresh R., Veltri E., Ezetimbe Study Group (2003). Effect of coadministration of ezetimibe and simvastatin on high-sensitivity C-reactive protein. Am. J. Cardiol..

[96] Pearson T.A., Ballantyne C.M., Veltri E., Shah A., Bird S., Lin J., Rosenberg E., Tershakovec A.M. (2009). Pooled analyses of effects on C-reactive protein and low density lipoprotein cholesterol in placebo-controlled trials of ezetimibe monotherapy or ezetimibe added to baseline statin therapy. Am. J. Cardiol..

[97] Pearson T., Ballantyne C., Sisk C., Shah A., Veltri E., Maccubbin D. (2007). Comparison of effects of ezetimibe/simvastatin versus simvastatin versus atorvastatin in reducing C-reactive protein and low-density lipoprotein cholesterol levels. Am. J. Cardiol..

[98] Liu P.Y., Liu Y.W., Lin L.J., Chen J.H., Liao J.K. (2009). Evidence for statin pleiotropy in humans: Differential effects of statins and ezetimibe on rho-associated coiled-coil containing protein kinase activity, endothelial function, and inflammation. Circulation.

[99] Suchy D., Łabuzek K., Stadnicki A., Okopień B. (2011). Ezetimibe—A new approach in hypercholesterolemia management. Pharmacol. Rep..

[100] Cho Y., Kim R.H., Park H., Wang H.J., Lee H., Kang E.S. (2020). Effect of Ezetimibe on Glucose Metabolism and Inflammatory Markers in Adipose Tissue. Biomedicines.

[101] Qin L., Yang Y.B., Yang Y.X., Zhu N., Li S.X., Liao D.F., Zheng X.L. (2014). Anti-inflammatory activity of ezetimibe by regulating NF-κB/MAPK pathway in THP-1 macrophages. Pharmacology.

[102] Ghanim H., Green K., Abuaysheh S., Patel R., Batra M., Chaudhuri A., Makdissi A., Kuhadiya N.D., Dandona P. (2017). Ezetimibe and simvastatin combination inhibits and reverses the pro-inflammatory and pro-atherogenic effects of cream in obese patients. Atherosclerosis.

[103] Rudofsky G., Reismann P., Groener J.B., Djuric Z., Fleming T., Metzner C., Grafe I.A., Bierhaus A., Nawroth P.P. (2012). Identical LDL-cholesterol lowering but non-identical effects on NF-κB activity: High dose simvastatin vs combination therapy with ezetimibe. Atherosclerosis.

[104] Landmesser U., Bahlmann F., Mueller M., Spiekermann S., Kirchhoff N., Schulz S., Manes C., Fischer D., de Groot K., Fliser D. (2005). Simvastatin versus ezetimibe: Pleiotropic and lipid-lowering effects on endothelial function in humans. Circulation.

[105] Al Badarin F.J., Kullo I.J., Kopecky S.L., Thomas R.J. (2009). Impact of ezetimibe on atherosclerosis: Is the jury still out?. Mayo. Clin. Proc..

[106] Araujo D.B., Bertolami M.C., Ferreira W.P., Abdalla D.S., Faludi A.A., Nakamura Y., Bricharello L.P. (2010). Pleiotropic effects with equivalent low-density lipoprotein cholesterol reduction: Comparative study between simvastatin and simvastatin/ezetimibe coadministration. J. Cardiovasc. Pharmacol..

[107] Kawagoe Y., Hattori Y., Nakano A., Aoki C., Tanaka S., Ohta S., Iijima T., Tomizawa A., Jojima T., Kase H. (2011). Comparative study between high-dose fluvastatin and low-dose fluvastatin and ezetimibe with regard to the effect on endothelial function in diabetic patients. Endocr. J..

[108] Ostad M.A., Eggeling S., Tschentscher P., Schwedhelm E., Boger R., Wenzel P., Meinertz T., Munzel T., Warnholtz A. (2009). Flowmediated dilation in patients with coronary artery disease is enhanced by high dose atorvastatin compared to combined low dose atorvastatin and ezetimibe: Results of the CEZAR study. Atherosclerosis.

[109] Gounari P., Tousoulis D., Antoniades C., Kampoli A.M., Stougiannos P., Papageorgiou N., Roulia G., Stefanadi E., Siasos G., Tsioufis C. (2010). Rosuvastatin but not ezetimibe improves endothelial function in patients with heart failure, by mechanisms independent of lipid lowering. Int. J. Cardiol..

[110] Fichtlscherer S., Schmidt-Lucke C., Bojunga S., Rössig L., Heeschen C., Dimmeler S., Zeiher A.M. (2006). Differential effects of short-term lipid lowering with ezetimibe and statins on endothelial function in patients with CAD: Clinical evidence for ‘pleiotropic’ functions of statin therapy. Eur. Heart J..

[111] Bulut D., Hanefeld C., Bulut-Streich N., Graf C., Mügge A., Spiecker M. (2005). Endothelial function in the forearm circulation of patients with the metabolic syndrome–effect of different lipid-lowering regimens. Cardiology.

[112] Bergen S.S., Van Itallie T.B., Tennent D.M., Sebrell W.H. (1959). Effect of an anion exchange resin on serum cholesterol in man. Proc. Soc. Exp. Biol. Med..

[113] Prawitt J., Staels B. (2010). Bile acid sequestrants: Glucose-lowering mechanisms. Metab. Syndr. Relat. Disord..

[114] Alder M., Bavishi A., Zumpf K., Peterson J., Stone N.J. (2020). A Meta-Analysis Assessing Additional LDL-C Reduction from Addition of a Bile Acid Sequestrant to Statin Therapy. Am. J. Med..

[115] Bays H.E., Davidson M., Jones M.R., Abby S.L. (2006). Effects of colesevelam hydrochloride on low-density lipoprotein cholesterol and high-sensitivity C-reactive protein when added to statins in patients with hypercholesterolemia. Am. J. Cardiol..

[116] Devaraj S., Autret B., Jialal I. (2006). Effects of colesevelam hydrochloride (WelChol) on biomarkers of inflammation in patients with mild hypercholesterolemia. Am. J. Cardiol..

[117] Zhou E., Hoeke G., Li Z., Eibergen A.C., Schonk A.W., Koehorst M., Boverhof R., Havinga R., Kuipers F., Coskun T. (2020). Colesevelam enhances the beneficial effects of brown fat activation on hyperlipidaemia and atherosclerosis development. Cardiovasc. Res..

[118] Jacobson T.A., Armani A., McKenney J.M., Guyton J.R. (2007). Safety considerations with gastrointestinally active lipid-lowering drugs. Am. J. Cardiol..

[119] Davidson M.H., Dillon M.A., Gordon B., Jones P., Samuels J., Weiss S., Isaacsohn J., Toth P., Burke S.K. (1999). Colesevelam hydrochloride (cholestagel): A new, potent bile acid sequestrant associated with a low incidence of gastrointestinal side effects. Arch. Intern. Med..

[120] Miller E.R., Pastor-Barriuso R., Dalal D., Riemersma R.A., Appel L.J., Guallar E. (2005). Meta-analysis: High-dosage vitamin E supplementation may increase all-cause mortality. Ann. Intern. Med..

[121] Jiang S., Pan Z., Li H., Li F., Song Y., Qiu Y. (2014). Meta-analysis: Low-dose intake of vitamin E combined with other vitamins or minerals may decrease all-cause mortality. J. Nutr. Sci. Vitaminol..

[122] Grundy S.M., Stone N.J., Bailey A.L., Beam C., Birtcher K.K., Blumenthal R.S., Braun L.T., de Ferranti S., Faiella-Tommasino J., Forman D.E. (2019). 2018 AHA/ACC/AACVPR/AAPA/ABC/ACPM/ADA/AGS/APhA/ASPC/NLA/PCNA Guideline on the Management of Blood Cholesterol: A Report of the American College of Cardiology/American Heart Association Task Force on Clinical Practice Guidelines. J. Am. Coll. Cardiol..

[123] Scherer D.J., Nelson A.J., Psaltis P.J., Nicholls S.J. (2017). Targeting low-density lipoprotein cholesterol with PCSK9 inhibitors. Intern. Med. J..

[124] Soffer D., Stoekenbroek R., Plakogiannis R. (2022). Small interfering ribonucleic acid for cholesterol lowering-Inclisiran: Inclisiran for cholesterol lowering. J. Clin. Lipidol..

[125] Tucker T.J., Embrey M.W., Alleyne C., Amin R.P., Bass A., Bhatt B., Bianchi E., Branca D., Bueters T., Buist N. (2021). A Series of Novel, Highly Potent, and Orally Bioavailable Next-Generation Tricyclic Peptide PCSK9 Inhibitors. J. Med. Chem..

[126] Toth S., Pella D., Fedacko J. (2020). Vaccines Targeting PSCK9 for the Treatment of Hyperlipidemia. Cardiol. Ther..

[127] Toth P.P., Bray S., Villa G., Palagashvili T., Sattar N., Stroes E.S.G., Worth G.M. (2022). Network Meta-Analysis of Randomized Trials Evaluating the Comparative Efficacy of Lipid-Lowering Therapies Added to Maximally Tolerated Statins for the Reduction of Low-Density Lipoprotein Cholesterol. J. Am. Heart Assoc..

[128] Sabatine M.S., Giugliano R.P., Keech A.C., Honarpour N., Wiviott S.D., Murphy S.A., Kuder J.F., Wang H., Liu T., Wasserman S.M. (2017). Evolocumab and Clinical Outcomes in Patients with Cardiovascular Disease. N. Engl. J. Med..

[129] Schwartz G.G., Steg P.G., Szarek M., Bhatt D.L., Bittner V.A., Diaz R., Edelberg J.M., Goodman S.G., Hanotin C., Harrington R.A. (2018). Alirocumab and Cardiovascular Outcomes after Acute Coronary Syndrome. N. Engl. J. Med..

[130] Ray K.K., Raal F.J., Kallend D.G., Jaros M.J., Koenig W., Leiter L.A., Landmesser U., Schwartz G.G., Lawrence D., Friedman A. (2023). Inclisiran and cardiovascular events: A patient-level analysis of phase III trials. Eur. Heart J..

[131] Li S., Zhang Y., Xu R.X., Guo Y.L., Zhu C.G., Wu N.Q., Qing P., Liu G., Dong Q., Li J.J. (2015). Proprotein convertase subtilisin-kexin type 9 as a biomarker for the severity of coronary artery disease. Ann. Med..

[132] Bohula E.A., Giugliano R.P., Leiter L.A., Verma S., Park J.G., Sever P.S., Lira Pineda A., Honarpour N., Wang H., Murphy S.A. (2018). Inflammatory and Cholesterol Risk in the FOURIER Trial. Circulation.

[133] Cannon C.P., Cariou B., Blom D., McKenney J.M., Lorenzato C., Pordy R., Chaudhari U., Colhoun H.M., Odyssey Combo II Investigators (2015). Efficacy and safety of alirocumab in high cardiovascular risk patients with inadequately controlled hypercholesterolaemia on maximally tolerated doses of statins: The ODYSSEY COMBO II randomized controlled trial. Eur. Heart J..

[134] Pradhan A.D., Aday A.W., Rose L.M., Ridker P.M. (2018). Residual Inflammatory Risk on Treatment with PCSK9 Inhibition and Statin Therapy. Circulation.

[135] Kühnast S., van der Hoorn J.W., Pieterman E.J., van den Hoek A.M., Sasiela W.J., Gusarova V., Peyman A., Schäfer H.L., Schwahn U., Jukema J.W. (2014). Alirocumab inhibits atherosclerosis, improves the plaque morphology, and enhances the effects of a statin. J. Lipid Res..

[136] Bernelot Moens S.J., Neele A.E., Kroon J., van der Valk F.M., Van den Bossche J., Hoeksema M.A., Hoogeveen R.M., Schnitzler J.G., Baccara-Dinet M.T., Manvelian G. (2017). PCSK9 monoclonal antibodies reverse the pro-inflammatory profile of monocytes in familial hypercholesterolaemia. Eur. Heart J..

[137] Tang Z., Jiang L., Peng J., Ren Z., Wei D., Wu C., Pan L., Jiang Z., Liu L. (2012). PCSK9 siRNA suppresses the inflammatory response induced by oxLDL through inhibition of NF-κB activation in THP-1-derived macrophages. Int. J. Mol. Med..

[138] Cheng J.M., Oemrawsingh R.M., Garcia-Garcia H.M., Boersma E., van Geuns R.J., Serruys P.W., Kardys I., Akkerhuis K.M. (2016). PCSK9 in relation to coronary plaque inflammation: Results of the ATHEROREMO-IVUS study. Atherosclerosis.

[139] Räber L., Ueki Y., Otsuka T., Losdat S., Häner J.D., Lonborg J., Fahrni G., Iglesias J.F., van Geuns R.J., Ondracek A.S. (2022). Effect of Alirocumab Added to High-Intensity Statin Therapy on Coronary Atherosclerosis in Patients with Acute Myocardial Infarction: The PACMAN-AMI Randomized Clinical Trial. JAMA.

[140] Nicholls S.J., Kataoka Y., Nissen S.E., Prati F., Windecker S., Puri R., Hucko T., Aradi D., Herrman J.R., Hermanides R.S. (2022). Effect of Evolocumab on Coronary Plaque Phenotype and Burden in Statin-Treated Patients Following Myocardial Infarction. JACC Cardiovasc. Imaging.

[141] Yano H., Horinaka S., Ishimitsu T. (2020). Effect of evolocumab therapy on coronary fibrous cap thickness assessed by optical coherence tomography in patients with acute coronary syndrome. J. Cardiol..

[142] Sugizaki Y., Otake H., Kawamori H., Toba T., Nagano Y., Tsukiyama Y., Yanaka K.I., Yamamoto H., Nagasawa A., Onishi H. (2020). Adding Alirocumab to Rosuvastatin Helps Reduce the Vulnerability of Thin-Cap Fibroatheroma: An ALTAIR Trial Report. JACC Cardiovasc. Imaging.

[143] Ako J., Hibi K., Tsujita K., Hiro T., Morino Y., Kozuma K., Shinke T., Otake H., Uno K., Louie M.J. (2019). Effect of Alirocumab on Coronary Atheroma Volume in Japanese Patients with Acute Coronary Syndrome—The ODYSSEY J-IVUS Trial. Circ. J..

[144] Lepor N.E., Sun J., Canton G., Contreras L., Hippe D.S., Isquith D.A., Balu N., Kedan I., Simonini A.A., Yuan C. (2021). Regression in carotid plaque lipid content and neovasculature with PCSK9 inhibition: A time course study. Atherosclerosis.

[145] Vlachopoulos C., Koutagiar I., Skoumas I., Terentes-Printzios D., Zacharis E., Kolovou G., Stamatelopoulos K., Rallidis L., Katsiki N., Bilianou H. (2019). Long-Term Administration of Proprotein Convertase Subtilisin/Kexin Type 9 Inhibitors Reduces Arterial FDG Uptake. JACC Cardiovasc. Imaging.

[146] Vecchié A., Bonaventura A., Meessen J., Novelli D., Minetti S., Elia E., Ferrara D., Ansaldo A.M., Scaravilli V., Villa S. (2021). PCSK9 is associated with mortality in patients with septic shock: Data from the ALBIOS study. J. Intern. Med..

[147] Genga K.R., Lo C., Cirstea M.S., Leitao Filho F.S., Walley K.R., Russell J.A., Linder A., Francis G.A., Boyd J.H. (2018). Impact of PCSK9 loss-of-function genotype on 1-year mortality and recurrent infection in sepsis survivors. EBioMedicine.

[148] Garshick M.S., Baumer Y., Dey A.K., Grattan R., Ng Q., Teague H.L., Yu Z.X., Chen M.Y., Tawil M., Barrett T.J. (2021). Characterization of PCSK9 in the Blood and Skin of Psoriasis. J. Investig. Dermatol..

[149] Luan C., Chen X., Zhu Y., Osland J.M., Gerber S.D., Dodds M., Hu Y., Chen M., Yuan R. (2019). Potentiation of Psoriasis-Like Inflammation by PCSK9. J. Investig. Dermatol..

[150] Leucker T.M., Weiss R.G., Schär M., Bonanno G., Mathews L., Jones S.R., Brown T.T., Moore R., Afework Y., Gerstenblith G. (2018). Coronary Endothelial Dysfunction is Associated with Elevated Serum PCSK9 Levels in People with HIV Independent of Low-Density Lipoprotein Cholesterol. J. Am. Heart Assoc..

[151] Zanni M.V., Stone L.A., Toribio M., Rimmelin D.E., Robinson J., Burdo T.H., Williams K., Fitch K.V., Lo J., Grinspoon S.K. (2017). Proprotein Convertase Subtilisin/Kexin 9 Levels in Relation to Systemic Immune Activation and Subclinical Coronary Plaque in HIV. Open Forum Infect. Dis..

[152] Boyd J.H., Fjell C.D., Russell J.A., Sirounis D., Cirstea M.S., Walley K.R. (2016). Increased Plasma PCSK9 Levels Are Associated with Reduced Endotoxin Clearance and the Development of Acute Organ Failures during Sepsis. J. Innate Immun..

[153] Sánchez-Pérez H., Quevedo-Abeledo J.C., Tejera-Segura B., de Armas-Rillo L., Rúa-Figueroa I., González-Gay M.A., Ferraz-Amaro I. (2020). Proprotein convertase subtilisin/kexin type 9 is related to disease activity and damage in patients with systemic erythematosus lupus. Ther. Adv. Musculoskelet. Dis..

[154] Liu A., Rahman M., Hafström I., Ajeganova S., Frostegård J. (2020). Proprotein convertase subtilisin kexin 9 is associated with disease activity and is implicated in immune activation in systemic lupus erythematosus. Lupus.

[155] Bhattacharya A., Chowdhury A., Chaudhury K., Shukla P.C. (2021). Proprotein convertase subtilisin/kexin type 9 (PCSK9): A potential multifaceted player in cancer. Biochim. Biophys. Acta Rev. Cancer.

[156] Bonaventura A., Vecchié A., Ruscica M., Grossi F., Dentali F. (2022). PCSK9 as a New Player in Cancer: New Opportunity or Red Herring?. Curr. Med. Chem..

[157] Momtazi-Borojeni A.A., Nik M.E., Jaafari M.R., Banach M., Sahebkar A. (2019). Potential anti-tumor effect of a nanoliposomal antiPCSK9 vaccine in mice bearing colorectal cancer. Arch. Med. Sci..

[158] Momtazi-Borojeni A.A., Nik M.E., Jaafari M.R., Banach M., Sahebkar A. (2019). Effects of immunization against PCSK9 in an experimental model of breast cancer. Arch. Med. Sci..

[159] Liu X., Bao X., Hu M., Chang H., Jiao M., Cheng J., Xie L., Huang Q., Li F., Li C.Y. (2020). Inhibition of PCSK9 potentiates immune checkpoint therapy for cancer. Nature.

[160] Banach M., Duell P.B., Gotto A.M., Laufs U., Leiter L.A., Mancini G.B.J., Ray K.K., Flaim J., Ye Z., Catapano A.L. (2020). Association of Bempedoic Acid Administration with Atherogenic Lipid Levels in Phase 3 Randomized Clinical Trials of Patients with Hypercholesterolemia. JAMA Cardiol..

[161] Samsoondar J.P., Burke A.C., Sutherland B.G., Telford D.E., Sawyez C.G., Edwards J.Y., Pinkosky S.L., Newton R.S., Huff M.W. (2017). Prevention of Diet-Induced Metabolic Dysregulation, Inflammation, and Atherosclerosis in Ldlr-/- Mice by Treatment with the ATP-Citrate Lyase Inhibitor Bempedoic Acid. Arterioscler. Thromb. Vasc. Biol..

[162] Burke A.C., Telford D.E., Sutherland B.G., Edwards J.Y., Sawyez C.G., Barrett P.H.R., Newton R.S., Pickering J.G., Huff M.W. (2018). Bempedoic Acid Lowers Low-Density Lipoprotein Cholesterol and Attenuates Atherosclerosis in Low-Density Lipoprotein Receptor-Deficient (LDLR+/- and LDLR-/-) Yucatan Miniature Pigs. Arterioscler. Thromb. Vasc. Biol..

[163] Nissen S.E., Lincoff A.M., Brennan D., Ray K.K., Mason D., Kastelein J.J.P., Thompson P.D., Libby P., Cho L., Plutzky J. (2023). Bempedoic Acid and Cardiovascular Outcomes in Statin-Intolerant Patients. N. Engl. J. Med..

[164] Cuchel M., Meagher E.A., du Toit Theron H., Blom D.J., Marais A.D., Hegele R.A., Averna M.R., Sirtori C.R., Shah P.K., Gaudet D. (2013). Efficacy and safety of a microsomal triglyceride transfer protein inhibitor in patients with homozygous familial hypercholesterolaemia: A single-arm, open-label, phase 3 study. Lancet.

[165] Blom D.J., Averna M.R., Meagher E.A., du Toit Theron H., Sirtori C.R., Hegele R.A., Shah P.K., Gaudet D., Stefanutti C., Vigna G.B. (2017). Long-Term Efficacy and Safety of the Microsomal Triglyceride Transfer Protein Inhibitor Lomitapide in Patients with Homozygous Familial Hypercholesterolemia. Circulation.

[166] Underberg J.A., Cannon C.P., Larrey D., Makris L., Blom D., Phillips H. (2020). Long-term safety and efficacy of lomitapide in patients with homozygous familial hypercholesterolemia: Five-year data from the Lomitapide Observational Worldwide Evaluation Registry (LOWER). J. Clin. Lipidol..

[167] D’Erasmo L., Steward K., Cefalù A.B., Di Costanzo A., Boersma E., Bini S., Arca M., van Lennep J.R., Italian and European Working Group on Lomitapide in HoFH (2022). Efficacy and safety of lomitapide in homozygous familial hypercholesterolaemia: The pan-European retrospective observational study. Eur. J. Prev. Cardiol..

[168] Blom D.J., Gaudet D., Hegele R.A., Patel D.S., Cegla J., Kolovou G., Marin L.M. (2022). A Case Series Assessing the Effects of Lomitapide on Carotid Intima-Media Thickness in Adult Patients with Homozygous Familial Hypercholesterolaemia in a Real-World Setting. Adv. Ther..

[169] Lee B., Park S.J., Lee S., Lee J., Lee E., Yoo E.S., Chung W.S., Sohn J.W., Oh B.C., Kim S. (2022). Lomitapide, a cholesterol-lowering drug, is an anticancer agent that induces autophagic cell death via inhibiting mTOR. Cell Death Dis..

[170] Sen P., Kandasamy T., Ghosh S.S. (2023). Multi-targeting TACE/ADAM17 and gamma-secretase of notch signalling pathway in TNBC via drug repurposing approach using Lomitapide. Cell. Signal..

[171] Raal F.J., Rosenson R.S., Reeskamp L.F., Hovingh G.K., Kastelein J.J.P., Rubba P., Ali S., Banerjee P., Chan K., Gipe D.A. (2020). Evinacumab for Homozygous Familial Hypercholesterolemia. N. Engl. J. Med..

[172] Stefanutti C., Chan D.C., Di Giacomo S., Morozzi C., Watts G.F. (2022). Long-Term Efficacy and Safety of Evinacumab in Patients with Homozygous Familial Hypercholesterolemia: Real-World Clinical Experience. Pharmaceuticals.

[173] Dewey F.E., Gusarova V., Dunbar R.L., O’Dushlaine C., Schurmann C., Gottesman O., McCarthy S., Van Hout C.V., Bruse S., Dansky H.M. (2017). Genetic and Pharmacologic Inactivation of ANGPTL3 and Cardiovascular Disease. N. Engl. J. Med..

[174] Reeskamp L.F., Nurmohamed N.S., Bom M.J., Planken R.N., Driessen R.S., van Diemen P.A., Luirink I.K., Groothoff J.W., Kuipers I.M., Knaapen P. (2021). Marked plaque regression in homozygous familial hypercholesterolemia. Atherosclerosis.

[175] Hussain A., Sun C., Selvin E., Nambi V., Coresh J., Jia X., Ballantyne C.M., Hoogeveen R.C. (2022). Triglyceride-rich lipoproteins, apolipoprotein C-III, angiopoietin-like protein 3, and cardiovascular events in older adults: Atherosclerosis Risk in Communities (ARIC) study. Eur. J. Prev. Cardiol..

[176] Sun T., Zhan W., Wei L., Xu Z., Fan L., Zhuo Y., Wang C., Zhang J. (2021). Circulating ANGPTL3 and ANGPTL4 levels predict coronary artery atherosclerosis severity. Lipids Health Dis..

[177] Lemoinne S.A., Pares A., Reig K., Ben Belkacem A.D., Kemgang Fankem F., Gaouar R., Poupon C., Housset C., Corpechot C., Chazouilleres O. (2018). Primary sclerosing cholangitis response to the combination of fbrates with ursodeoxycholic acid: French-Spanish experience. Clin. Res. Hepatol. Gastroenterol..

[178] Zhang H., Li S., Feng Y., Zhang Q., Xie B. (2022). Efficacy of fibrates in the treatment of primary biliary cholangitis: A meta-analysis. Clin. Exp. Med..

[179] Das Pradhan A., Glynn R.J., Fruchart J.C., MacFadyen J.G., Zaharris E.S., Everett B.M., Campbell S.E., Oshima R., Amarenco P., Blom D.J. (2022). Triglyceride Lowering with Pemafibrate to Reduce Cardiovascular Risk. N. Engl. J. Med..

[180] Shinozaki S., Tahara T., Lefor A.K., Ogura M. (2020). Pemafibrate decreases markers of hepatic inflammation in patients with non-alcoholic fatty liver disease. Clin. Exp. Hepatol..

[181] Visseren F.L.J., Mach F., Smulders Y.M., Carballo D., Koskinas K.C., Bäck M., Benetos A., Biffi A., Boavida J.M., Capodanno D. (2021). 2021 ESC Guidelines on cardiovascular disease prevention in clinical practice. Eur. Heart J..

[182] Montaigne D., Butruille L., Staels B. (2021). PPAR control of metabolism and cardiovascular functions. Nat. Rev. Cardiol..

[183] Bougarne N., Weyers B., Desmet S.J., Deckers J., Ray D.W., Staels B., De Bosscher K. (2018). Molecular Actions of PPARα in Lipid Metabolism and Inflammation. Endocr. Rev..

[184] Kytikova O.Y., Perelman J.M., Novgorodtseva T.P., Denisenko Y.K., Kolosov V.P., Antonyuk M.V., Gvozdenko T.A. (2020). Peroxisome Proliferator-Activated Receptors as a Therapeutic Target in Asthma. PPAR Res..

[185] Botta M., Audano M., Sahebkar A., Sirtori C.R., Mitro N., Ruscica M. (2018). PPAR agonists and metabolic syndrome: An established role?. Int. J. Mol. Sci..

[186] Banno A., Reddy A.T., Lakshmi S.P., Reddy R.C. (2018). PPARs: Key regulators of airway inflammation and potential therapeutic targets in asthma. Nucl. Recept. Res..

[187] Nobs S.P., Natali S., Pohlmeier L., Okreglicka K., Schneider C., Kurrer M., Sallusto F., Kopf M. (2017). PPARγ in dendritic cells and T cells drives pathogenic type-2 effector responses in lung inflammation. J. Exp. Med..

[188] Krönke G., Kadl A., Ikonomu E., Blüml S., Fürnkranz A., Sarembock I.J., Bochkov V.N., Exner M., Binder B.R., Leitinger N. (2007). Expression of heme oxygenase-1 in human vascular cells is regulated by peroxisome proliferator-activated receptors. Arterioscler. Thromb. Vasc. Biol..

[189] Rommelaere S., Millet V., Gensollen T., Bourges C., Eeckhoute J., Hennuyer N., Baugé E., Chasson L., Cacciatore I., Staels B. (2013). PPARalpha regulates the production of serum Vanin-1 by liver. FEBS Lett..

[190] Muhlestein J.B., May H.T., Jensen J.R., Horne B.D., Lanman R.B., Lavasani F., Wolfert R.L., Pearson R.R., Yannicelli H.D., Anderson J.L. (2006). The reduction of inflammatory biomarkers by statin, fibrate, and combination therapy among diabetic patients with mixed dyslipidemia: The DIACOR (Diabetes and Combined Lipid Therapy Regimen) study. J. Am. Coll. Cardiol..

[191] Toyoda T., Kamei Y., Kato H., Sugita S., Takeya M., Suganami T., Ogawa Y. (2008). Effect of peroxisome proliferator-activated receptor-alpha ligands in the interaction between adipocytes and macrophages in obese adipose tissue. Obesity.

[192] Mazzon E., Cuzzocrea S. (2007). Absence of functional peroxisome proliferator-activated receptor-alpha enhanced ileum permeability during experimental colitis. Shock.

[193] Elaidy S.M., Essawy S.S., Hussain M.A., El-Kherbetawy M.K., Hamed E.R. (2018). Modulation of the IL-23/IL-17 axis by fenofibrate ameliorates the ovalbumin/lipopolysaccharide-induced airway inflammation and bronchial asthma in rats. Naunyn. Schmiedebergs Arch. Pharmacol..

[194] Paw M., Wnuk D., Kądziołka D., Sęk A., Lasota S., Czyż J., Madeja Z., Michalik M. (2018). Fenofibrate Reduces the Asthma-Related Fibroblast-To-Myofibroblast Transition by TGF-Β/Smad2/3 Signaling Attenuation and Connexin 43-Dependent Phenotype Destabilization. Int. J. Mol. Sci..

[195] Chen Y., Hu Y., Lin M., Jenkins A.J., Keech A.C., Mott R., Lyons T.J., Ma J.X. (2013). Therapeutic effects of PPARalpha agonists on diabetic retinopathy in type diabetes models. Diabetes.

[196] Arai H., Yamashita S., Yokote K., Araki E., Suganami H., Ishibashi S., K-877 Study Group (2018). Efficacy and Safety of Pemafibrate versus Fenofibrate in Patients with High Triglyceride and Low HDL Cholesterol Levels: A Multicenter, Placebo-Controlled, Double-Blind, Randomized Trial. J. Atheroscler. Thromb..

[197] Giampietro L., Ammazzalorso A., Amoroso R., De Filippis B. (2019). Development of Fibrates as Important Scaffolds in Medicinal Chemistry. ChemMedChem.

[198] Virani S.S. (2022). The Fibrates Story—A Tepid End to a Prominent Drug. N. Engl. J. Med..

[199] Bradberry J.C., Hilleman D.E. (2013). Overview of omega-3 Fatty Acid therapies. Pharm. Ther..

[200] Drenjančević I., Pitha J. (2022). Omega-3 Polyunsaturated Fatty Acids-Vascular and Cardiac Effects on the Cellular and Molecular Level (Narrative Review). Int. J. Mol. Sci..

[201] Bornfeldt K.E. (2021). Triglyceride lowering by omega-3 fatty acids: A mechanism mediated by N-acyl taurines. J. Clin. Investig..

[202] Nettleton J.A. (1995). Omega-3 Fatty Acids and Health.

[203] Surette M.E. (2008). The science behind dietary omega-3 fatty acids. Can. Med. Assoc. J..

[204] Natto Z.S., Yaghmoor W., Alshaeri H.K., Van Dyke T.E. (2019). Omega-3 Fatty Acids Effects on Inflammatory Biomarkers and Lipid Profiles among Diabetic and Cardiovascular Disease Patients: A Systematic Review and Meta-Analysis. Sci. Rep..

[205] Bays H., Kwiterovich P.O. (2010). Fish oils in the treatment of dyslipidemia and cardiovascular disease. The Johns Hopkins Textbook of Dyslipidemia.

[206] Bhatt D.L. (2020). Mechanisms of action, efficacy, and safety of icosapent ethyl: From bench to bedside. Eur. Heart J. Suppl..

[207] Bays H.E., Braeckman R.A., Ballantyne C.M., Kastelein J.J., Otvos J.D., Stirtan W.G., Soni P.N. (2012). Icosapent ethyl, a pure EPA omega-3 fatty acid: Effects on lipoprotein particle concentration and size in patients with very high triglyceride levels (the MARINE study). J. Clin. Lipidol..

[208] Ballantyne C.M., Bays H.E., Kastelein J.J., Stein E., Isaacsohn J.L., Braeckman R.A., Soni P.N. (2012). Efficacy and safety of eicosapentaenoic acid ethyl ester (AMR101) therapy in statin-treated patients with persistent high triglycerides (from the ANCHOR study). Am. J. Cardiol..

[209] Bhatt D.L., Steg P.G., Miller M., Brinton E.A., Jacobson T.A., Ketchum S.B., Doyle R.T., Juliano R.A., Jiao L., Granowitz C. (2019). Effects of Icosapent Ethyl on Total Ischemic Events: From REDUCE-IT. J. Am. Coll. Cardiol..

[210] Granger C.B., Nelson A.J., Pagidipati N.J. (2019). Risk of Total Events with Icosapent Ethyl: Can We Reduce It?. J. Am. Coll. Cardiol..

[211] Gaba P., Bhatt D.L., Steg P.G., Miller M., Brinton E.A., Jacobson T.A., Ketchum S.B., Juliano R.A., Jiao L., Doyle R.T. (2022). Prevention of Cardiovascular Events and Mortality with Icosapent Ethyl in Patients with Prior Myocardial Infarction. J. Am. Coll. Cardiol..

[212] Peterson B.E., Bhatt D.L., Steg P.G., Miller M., Brinton E.A., Jacobson T.A., Ketchum S.B., Juliano R.A., Jiao L., Doyle R.T. (2022). Treatment with Icosapent Ethyl to Reduce Ischemic Events in Patients with Prior Percutaneous Coronary Intervention: Insights from REDUCE-IT PCI. J. Am. Heart Assoc..

[213] Verma S., Bhatt D.L., Steg P.G., Miller M., Brinton E.A., Jacobson T.A., Dhingra N.K., Ketchum S.B., Juliano R.A., Jiao L. (2021). Icosapent Ethyl Reduces Ischemic Events in Patients with a History of Previous Coronary Artery Bypass Grafting: REDUCE-IT CABG. Circulation.

[214] Majithia A., Bhatt D.L., Friedman A.N., Miller M., Steg P.G., Brinton E.A., Jacobson T.A., Ketchum S.B., Juliano R.A., Jiao L. (2021). Benefits of Icosapent Ethyl across the Range of Kidney Function in Patients with Established Cardiovascular Disease or Diabetes: REDUCE-IT RENAL. Circulation.

[215] Ridker P.M., Rifai N., MacFadyen J., Glynn R.J., Jiao L., Steg P.G., Miller M., Brinton E.A., Jacobson T.A., Tardif J.C. (2022). Effects of Randomized Treatment with Icosapent Ethyl and a Mineral Oil Comparator on Interleukin-1β, Interleukin-6, C-Reactive Protein, Oxidized Low-Density Lipoprotein Cholesterol, Homocysteine, Lipoprotein(a), and Lipoprotein-Associated Phospholipase A2: A REDUCE-IT Biomarker Substudy. Circulation.

[216] Budoff M.J., Bhatt D.L., Kinninger A., Lakshmanan S., Muhlestein J.B., Le V.T., May H.T., Shaikh K., Shekar C., Roy S.K. (2020). Effect of icosapent ethyl on progression of coronary atherosclerosis in patients with elevated triglycerides on statin therapy: Final results of the EVAPORATE trial. Eur. Heart J..

[217] Abdelsameea A.A., Alsemeh A.E., Alabassery N., Samy W., Fawzy A., Abbas N.A.T. (2023). Icosapent ethyl alleviates acetic acid-induced ulcerative colitis via modulation of SIRT1 signaling pathway in rats. Int. Immunopharmacol..

[218] Osadnik T., Goławski M., Lewandowski P., Morze J., Osadnik K., Pawlas N., Lejawa M., Jakubiak G.K., Mazur A., Schwingschackl L. (2022). A network meta-analysis on the comparative effect of nutraceuticals on lipid profile in adults. Pharmacol. Res..

[219] Cicero A.F., Derosa G., Parini A., Maffioli P., D’Addato S., Reggi A., Giovannini M., Borghi C. (2013). Red yeast rice improves lipid pattern, high-sensitivity C-reactive protein, and vascular remodeling parameters in moderately hypercholesterolemic Italian subjects. Nutr. Res..

[220] Domenech M., Casas R., Ruiz-León A.M., Sobrino J., Ros E., Estruch R. (2019). Effects of a Novel Nutraceutical Combination (Aquilea Colesterol^®^) on the Lipid Profile and Inflammatory Biomarkers: A Randomized Control Trial. Nutrients.

[221] Protic O., Di Pillo R., Montesanto A., Galeazzi R., Matacchione G., Giuliani A., Sabbatinelli J., Gurău F., Silvestrini A., Olivieri F. (2022). Randomized, Double-Blind, Placebo-Controlled Trial to Test the Effects of a Nutraceutical Combination Monacolin K-Free on the Lipid and Inflammatory Profile of Subjects with Hypercholesterolemia. Nutrients.

[222] Bumrungpert A., Lilitchan S., Tuntipopipat S., Tirawanchai N., Komindr S. (2018). Ferulic Acid Supplementation Improves Lipid Profiles, Oxidative Stress, and Inflammatory Status in Hyperlipidemic Subjects: A Randomized, Double-Blind, Placebo-Controlled Clinical Trial. Nutrients.

[223] Emamat H., Zahedmehr A., Asadian S., Nasrollahzadeh J. (2022). The effect of barberry (*Berberis integerrima*) on lipid profile and systemic inflammation in subjects with cardiovascular risk factors: A randomized controlled trial. BMC Complement. Med. Ther..

[224] Adorni M.P., Zimetti F., Lupo M.G., Ruscica M., Ferri N. (2020). Naturally Occurring PCSK9 Inhibitors. Nutrients.

[225] Kooshki A., Tofighiyan T., Miri M. (2019). A synbiotic supplement for inflammation and oxidative stress and lipid abnormalities in hemodialysis patients. Hemodial. Int..

[226] Laffin L.J., Bruemmer D., Garcia M., Brennan D.M., McErlean E., Jacoby D.S., Michos E.D., Ridker P.M., Wang T.Y., Watson K.E. (2023). Comparative Effects of Low-Dose Rosuvastatin, Placebo, and Dietary Supplements on Lipids and Inflammatory Biomarkers. J. Am. Coll. Cardiol..

